# Gene-level gut microbiome signatures as predictive biomarkers for response to immune checkpoint inhibitors across multiple cancer types

**DOI:** 10.1080/19490976.2026.2662690

**Published:** 2026-04-23

**Authors:** Fengyun Zhang, Kaimiao Hu, Changming Sun, Ruibing Chen, Guangjian Ni, Xiaofeng Liu, Leyi Wei, Ran Su

**Affiliations:** a College of Intelligence and Computing, Tianjin University, Tianjin, China; b CSIRO Data61, Epping, NSW, Australia; c School of Pharmaceutical Science and Technology, Faculty of Medicine, Tianjin University, Tianjin, China; d Academy of Medical Engineering and Translational Medicine, Tianjin University, Tianjin, China; e Haihe Laboratory of Brain-Computer Interaction and Human-Machine Integration, Tianjin, China; f State Key Laboratory of Advanced Medical Materials and Devices, Tianjin, China; g Tianjin Key Laboratory of Brain Science and Neuroengineering, Tianjin, China; h The First Department of Breast Cancer, Key Laboratory of Breast Cancer Prevention and Therapy, Ministry of Education, Tianjin Medical University Cancer Institute and Hospital, Tianjin, China; i National Clinical Research Center for Cancer, Key Laboratory of Cancer Prevention and Therapy, Tianjin's Clinical Research Center for Cancer, Tianjin, China; j Centre for Artificial Intelligence driven Drug Discovery, Faculty of Applied Science, Macao Polytechnic University, Macao, SAR, China

**Keywords:** Immune checkpoint inhibitors, predictive biomarkers, deep learning, biological prior knowledge, combination immune checkpoint blockade

## Abstract

Targeting programmed cell death protein 1 (PD-1) and cytotoxic T-lymphocyte-associated protein 4 (CTLA-4) with immune checkpoint inhibitors (ICIs) has improved survival across multiple cancer types, but the variability in patient response highlights the need for better predictive biomarkers. Existing studies rely on taxonomic abundance derived from reference genome databases, limiting the discovery and functional interpretation of uncharacterized microbes. Here, we integrated metagenomic data from multiple ICI-treated cohorts spanning diverse cancer types and geographic regions and developed a deep learning model, named BioP-VAE, that incorporates biological prior knowledge via protein sequence embeddings and uses gene-level microbial abundance features as input. Gene-level microbial abundance outperformed taxonomy abundance in predicting both ICI response and 12-month progression-free survival (PFS). In patients receiving combination immune checkpoint blockade (CICB), BioP-VAE achieved a mean AUC of 0.89 in intracohort and 0.88 in cross-cohort evaluation. Notably, in the monotherapy-treated intracohorts, BioP-VAE achieved a mean AUC of 0.97. Feature attribution analysis revealed key microbial genes. Additionally, we identified distinct predictive microbial signatures via age-stratified analysis, suggesting that host age may modulate microbiome‒immune interactions. Importantly, this is the first large-scale study to evaluate gene-level microbial abundance features for ICI response prediction across multiple cancer types by deep learning. Our findings demonstrate that incorporating biological prior knowledge into deep learning models can improve the discovery of microbial biomarkers that can be generalized across cancer types and treatment settings, offering a novel strategy for patient stratification in immunotherapy.

## Introduction

Immune checkpoint blockade (ICB) therapy has achieved significant progress in treating various malignancies by reversing tumor-induced immune suppression,^1-3^ demonstrating promising efficacy in cancers such as melanoma, liver cancer, and lung cancer.^4^ Combination ICB (CICB) targeting both programmed cell death protein 1 (PD-1) and cytotoxic T-lymphocyte-associated protein 4 (CTLA-4) has demonstrated synergistic antitumor activity. With the continued expansion of ICB indications,^5^ the CICB has been increasingly applied in a broader range of cancers, such as melanoma,^6^ mesothelioma,^7^ and hepatocellular carcinoma.^8^ However, the clinical response to ICB remains highly heterogeneous across patients, with durable clinical benefits observed in only 20%–60% of cases.^9^ Despite reported age-associated differences in ICB response, the clinical evidence remains inconsistent.^10-12^ These differences may be attributed to age-related immune remodeling, including reduced T cell activity and the upregulation of exhaustion markers, as well as the decline in gut microbial diversity and compositional shifts associated with aging.^13^ Therefore, pretreatment identification of potential responders and the development of robust biomarkers to stratify patients are imperative for tailoring personalized therapeutic strategies.

Recent studies have attempted to develop various predictive biomarkers, including tumor mutational burden (TMB),^14^ PD-L1 expression levels, and tumor-infiltrating lymphocytes. However, these tumor tissue-dependent “invasive” biomarkers are limited by patient compliance,^15^ the difficulty of monitoring dynamic changes over time. The gut microbiome has garnered increasing attention as a “tumor-extrinsic” predictive biomarker. For example, Pascal et al. developed a diagnostic model based on microbial species abundance, achieving a sensitivity of 81.8% for Crohn's disease (CD).^16^ Goallec et al. proposed a computational framework to infer microbiome-derived features for host phenotype prediction.^17^ Recent research has revealed significant correlations between the gut microbial composition and immune checkpoint inhibitor (ICI) response.^18-20^ However, most existing studies for predicting ICI response rely on alignment-based taxonomic annotation methods,^21^^,^^22^ which are dependent on microbial reference genome databases. This dependence not only limits taxonomic resolution and excludes novel or unclassified microorganisms with potential biological relevance but also overlooks the substantial genetic and functional heterogeneity that may exist even within the same microbial species.^23^ Moreover, there is a lack of consensus regarding the specific microbial taxa identified across studies, with limited reproducibility across cohorts and cancer types.

Several recent studies have examined microbial gene-level and function-level signatures in relation to ICI response. Cai et al. integrated metagenomic data across multiple cohorts to identify microbial gene markers and functional pathways, highlighting potential mechanistic links between the gut microbiome and antitumor immunity.^24^ Dora et al. applied metatranscriptomic analyses in ICI-treated non-small cell lung cancer (NSCLC) patients to identify RNA-based microbial signatures that distinguish clinical outcomes.^25^ Despite these advances, the predictive potential of gene-level microbial features across different cohorts and treatment settings remains insufficiently explored. Previous efforts to leverage machine learning for ICI response prediction have often been constrained by coarse-grained feature extraction and limited model generalizability. Consequently, there is a critical necessity to integrate high-resolution microbiome data with expressive modeling frameworks to enhance predictive performance and biological interpretability.

In this study, we constructed predictive models for ICI response using fecal metagenomic sequencing samples and evaluated the predictive performance of multiple types of features. We first integrated four publicly available fecal metagenomic cohorts, encompassing ICB-treated patients from diverse geographic regions and cancer types. Our study directly extracted gene-level microbial abundance features from metagenomes as model inputs. Furthermore, we developed a novel ICI response prediction model called BioP-VAE based on a variational autoencoder (VAE)^26^ deep learning framework. We also analyzed the feature attribution scores of the BioP-VAE model between responders and non-responders to further elucidate the biological relevance of key predictive features and performed downstream pathway and functional enrichment analyses to uncover potential biological mechanisms underlying the treatment response. Overall, our findings demonstrate that gene-level microbial abundance outperforms microbial taxonomic abundance features in predicting ICI response. Specifically, gene-level microbial features effectively predict treatment outcomes across multiple cancer types and cohorts in patients receiving CICB, whereas this predictive capacity is not observed in patients treated with anti-PD-1/anti-CTLA-4 monotherapy, indicating a treatment-specific relationship between the microbiome and therapeutic response.

## Results

### Study design

**Figure 1.**
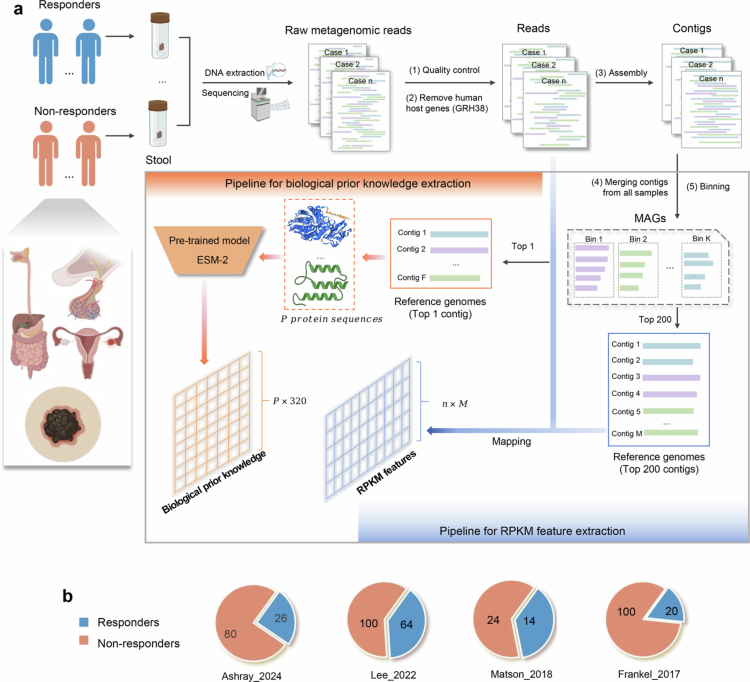
Overall workflow and proportion of ICI therapy responders. (a) Computational workflow for feature extraction (created using BioRender.com). The raw metagenomic reads were subjected to quality control and filtered to remove human host sequences (GRCh38^27^), followed by assembly into contigs. Contigs from all the samples were merged and binned. For each bin, the first contig was selected as the input for biological prior knowledge feature extraction, while the top 200 longest contigs were used as reference genomes for calculating RPKM features. The final top 1 and top 200 reference genomes contained 1269 and 161,107 contigs. In the pipeline for extracting biological prior knowledge, reference genomes (top 1 contigs) were processed using the tool Prodigal for protein sequence prediction, and protein-level representations were then extracted using the pretrained ESM-2^28^ model. In the pipeline for RPKM feature extraction, sample reads are mapped to non-redundant reference genomes (top 200 contigs) to measure the gene-level microbial abundance of genomic fragments. To ensure consistency in feature dimensionality across all cohorts, the reference genomes were fixed based on the Ashray_2024 cohort. (b) Pie charts illustrating the proportions of ICI responders and non-responders. Published studies are denoted by “author_year”.

In this study, we developed a novel prediction model called BioP-VAE (Supplementary Figure 1 illustrates the model architecture) and investigated the association between the gut microbiome composition and the clinical response to ICI and 12-month progression-free survival (PFS) in cancer patients, leveraging gene-level microbial abundance features quantified by RPKM (reads per kilobase per million mapped reads), also referred to as RPKM features. The overall workflow of metagenomic feature extraction is shown in Figure 1a. The RPKM features and biological prior knowledge representations served as input to BioP-VAE.

We collected four fecal shotgun metagenomics datasets from published studies. All patients received ICI therapy. The Ashray_2024 cohort^21^ comprised patients with histologically confirmed advanced rare solid tumors, all of whom were treated with CICB targeting both PD-1 and CTLA-4. Specifically, the tumor types included upper gastrointestinal and biliary cancers (UGB, 36%), neuroendocrine neoplasms (NEN, 34%), and rare gynecological tumors (GYN, 30%). The Lee_2022,^29^ Matson_2018,^30^ and Frankel_2017^31^ cohorts comprised patients with melanoma (MEL). All patients in the Lee_2022 and Matson_2018 cohorts received anti-PD-1 therapy. In the Frankel_2017 cohort, 62% of patients were treated with CICB, and the remaining 38% received either anti-PD-1 or anti-CTLA-4 therapy. The proportion of responders and non-responders to ICI therapy is summarized in Figure 1b.

### Gene-level microbial abundance features outperform taxonomic profiles in predicting ICI response

To evaluate the impact of feature types and selection strategies on model performance, we performed four comparative experiments. The performance metrics were averaged across fivefold cross-validation to derive the final results for each feature set. Furthermore, we employed the integrated gradient (IG)^32^ method to interpret the model predictions and quantify the contribution of individual features.

**Figure 2.**
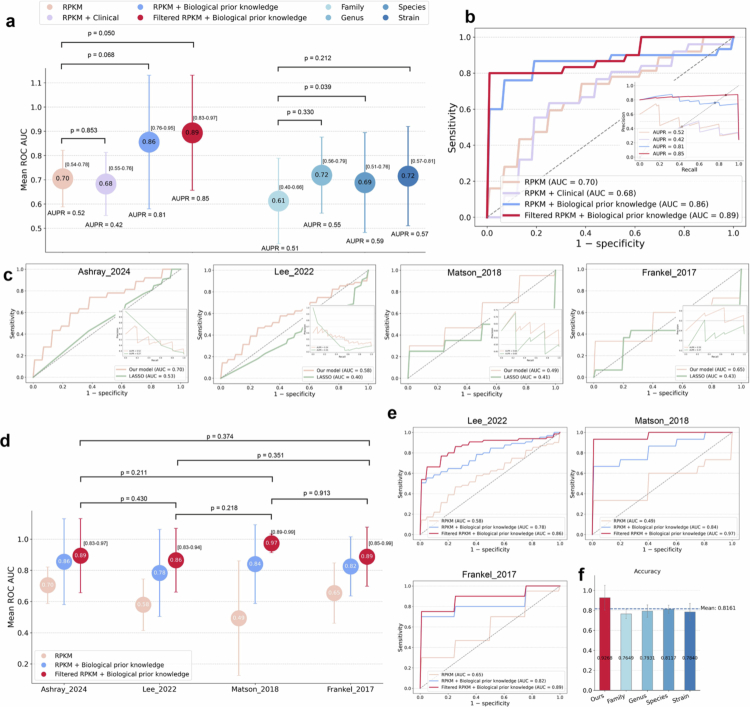
Performance comparison between gene-level microbial abundance and taxonomic abundance features. (a) Predictive performance of ICI response across various combinations of RPKM features, taxonomic abundance, and selection strategies in the Ashray_2024 cohort. The circles indicate the mean AUC scores, with error bars representing the standard deviation (SD). The AUPR values are also shown. The statistical significance between AUC scores within identical feature categories is indicated by p values. The bracketed values denote 95% bootstrap confidence intervals (1000 replicates). (b) Receiver operating characteristic (ROC) curves corresponding to the AUC results in (a), with the inset showing the corresponding precision‒recall (PR) curves. (c) Comparison of ROC curves between the BioP-VAE model and the LASSO classifier using all RPKM features. The inset panels display the corresponding PR curves. (d) Predictive performance of ICI response in different cohorts. (e) ROC curves corresponding to the AUC results are shown in (d). (f) Performance comparison based on accuracy in the Ashray_2024 cohort. The panel shows the metric scores for five feature types: filtered RPKM features with biological prior knowledge (ours) and four taxonomic abundance features at the family, genus, species, and strain levels. The bars represent the mean values from fivefold cross-validation, with the error bars indicating the standard deviation. The dashed lines indicate the overall average accuracy across all feature types.

First, we assessed the contribution of clinical factors to ICI response prediction by comparing model performance using RPKM features versus a combination of RPKM features and 15 potentially relevant clinical factors (see Methods) in the Ashray_2024 cohort. Unexpectedly, the integration of clinical factors marginalized the predictive performance, with the mean area under the receiver operating characteristic curve (AUC) decreasing from 0.70 to 0.68 (*p* = 0.853; Figure 2a and b). Although previous studies observed that a low blood albumin and high neutrophil-to-lymphocyte ratio (NLR) are associated with clinical progressive disease (cPD),^33^^,^^34^ these commonly measured clinical factors provide limited information for distinguishing treatment responders from non-responders and are useful for identifying patients with the worst prognosis. To explore the limited impact of clinical variables on model performance, we generated partial dependence plots (PDPs, Supplementary Figure 12) for seven numerical clinical factors. The PDP curves showed irregular, non-monotonic patterns across the observed value ranges, indicating weak marginal effects on the predictions. Moreover, RPKM features dominate the input space with over 160,000 dimensions, while clinical variables are comparatively few. Using the same RPKM features, our model also outperformed the baseline LASSO classifier across all four cohorts (Figure 2c).

Second, previous studies have suggested that incorporating protein sequence-derived information can enhance model performance in biomedical prediction tasks.^35-37^ Motivated by this, we incorporated an attention mechanism to introduce protein-level features as biological prior knowledge, guiding the model's learning of RPKM features. The results showed a notable improvement, with the average AUC score in the Ashray_2024 cohort increasing from 0.70 to 0.86 (*p* = 0.068; Figure 2a and b). Similar improvements were consistently observed across all melanoma cohorts, with the Matson_2018 cohort showing an AUC increase of over 35% (Figure 2d and e). These findings indicate that incorporating protein-level biological prior knowledge can help compensate for information gaps in RPKM features.

Third, given that contigs within the same bin may contain highly homologous sequences, using all RPKM features could introduce redundancy information. To address this issue, we applied a nonlinear feature selection strategy based on mutual information (MI).^38^ Specifically, we selected the top 80,000 RPKM features ranked by their MI scores (the cumulative mutual information contribution curves are shown in Supplementary Figure 11). The results demonstrated that MI-based feature selection improved model performance (Figure 2a and d). In particular, in the Matson_2018 cohort, the AUC score increased from 0.84 to 0.97 (Figure 2d and e).

Fourth, traditional microbiome signature construction relies on taxonomic abundance profiles, which are inherently limited by reference databases and may fail to capture uncharacterized species potentially critical to host immunotherapy responses. In contrast, RPKM features directly reflect the microbial gene expression activity, offering sensitivity and generalizability for detecting microbiota‒host interactions. To validate this advantage, we systematically compared the predictive performance of filtered RPKM features integrated with biological prior knowledge against four types of taxonomic abundance features (at the family, genus, species, and strain levels) in the Ashray_2024 cohort. The results demonstrated that the RPKM features outperformed all the taxonomic abundance features, achieving an accuracy of 92.68% (Figure 2f) and an AUC of 0.89 (Figure 2a and b). The strain-level features yielded the best performance, with a maximum AUC of 0.72 (Figure 2a). Given the class imbalance in the Ashray_2024 cohort, we further assessed model performance using the area under the precision‒recall curve (AUPR). Notably, the RPKM features exhibited a markedly higher AUPR of 0.85, whereas all taxonomic abundance features yielded AUPR values below 0.60 (as shown in Figure 2a).

**Figure 3.**
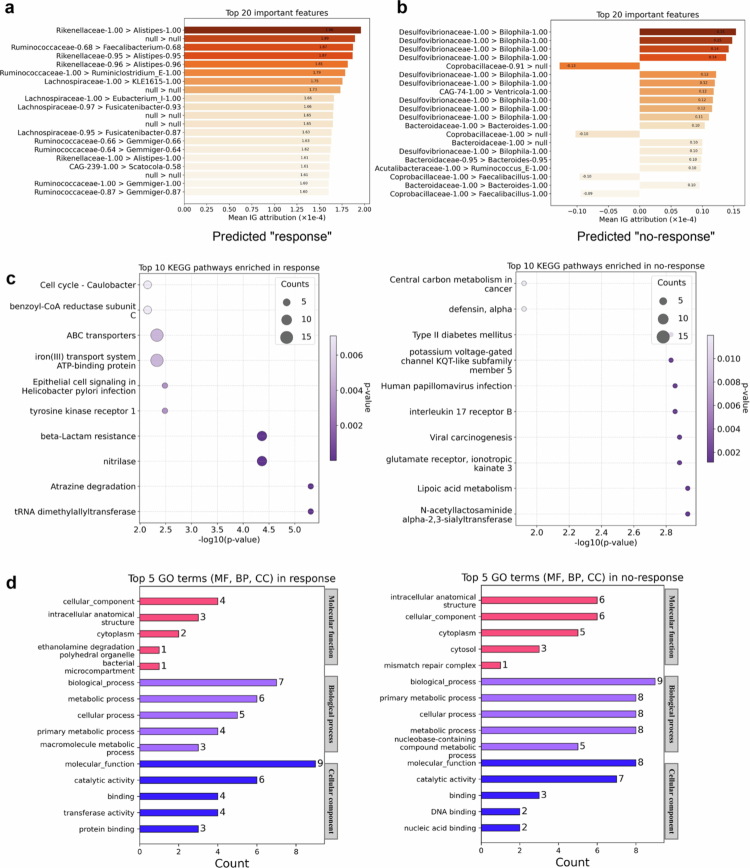
The IG attribution analysis in the Ashray_2024 cohort. The analysis was performed on filtered RPKM features integrated with biological prior knowledge. IG scores were calculated from the validation sets of fivefold cross-validation, averaging the attribution values from correctly predicted samples across all folds. The feature (contig) name/label was annotated according to the GTDB database.^39^ (a and b) Top 20 features ranked by the mean IG values. The y-axis labels represent features in the format “FamilyName-confidence > GenusName-confidence” (e.g., “Rikenellaceae-1.00 > Alistipes-1.00”), with the corresponding IG values displayed on the x-axis. The absence of family or genus annotations (or both) in certain cases indicates that the corresponding contig could not be taxonomically classified based on the GTDB, and such cases are denoted as “null.” (c) Top 10 KEGG pathway enrichment. Bubble size represents the gene count, the bubble color represents the *p*-value, and the x-axis shows −log10 (*p*-value). (d) The top 5 GO terms count in the three categories (molecular function, biological process, and cellular component).

Finally, we applied the IG algorithm to assign attribution scores to input features in correctly predicted samples, aiming to better understand the decision basis using filtered RPKM features with biological prior knowledge. Each feature corresponds to a contig, which was taxonomically annotated using the Genome Taxonomy Database (GTDB).^39^ Based on the taxonomic annotations, we summarized potential associations between differences in RPKM features and response to ICI therapy. In the Ashray_2024 cohort, Figure 3a and b present the top 20 RPKM features with the highest absolute contribution scores in predicted responders and non-responders. The high positive contribution features in responders were predominantly derived from the Rikenellaceae, Ruminococcaceae, and Lachnospiraceae families. Notably, the high attribution scores assigned to Ruminococcaceae in responders are consistent with previous studies on metastatic melanoma, which found that patients who responded to anti-PD-1 therapy exhibited higher abundances of the Ruminococcaceae family, including the *Faecalibacterium* genus.^19^^,^^30^^,^^40^ In non-responders, the Desulfovibrionaceae and Bacteroidaceae families showed major positive IG scores, while the Coprobacillaceae family contributed negative IG scores. Consistently, Thomas et al. reported that the pro-inflammatory Desulfovibrionaceae family was enriched in renal cancer patients and its abundance significantly decreased following effective PD-1 blockade therapy, suggesting that this bacterial family may impair ICI efficacy by maintaining intestinal inflammation.^41^

Furthermore, we performed Kyoto Encyclopedia of Genes and Genomes (KEGG) and Gene Ontology (GO) enrichment analyses on the top 20 important contigs. As shown in Figure 3c, the KEGG pathway analysis revealed a significant enrichment of nitrilase in the response group. Nitrilase, a member of the Nit2 family, functions primarily to remove potentially toxic intermediates in nitrogen metabolism.^42^ Nitrilase has been implicated in T cell regulation, with studies showing that Nit1 deficiency leads to T cell hyperproliferation and enhanced activation upon TCR stimulation.^43^ Recent evidence also suggests that NIT2 can restrain oxidative phosphorylation in cancer cells.^44^ Given these immunomodulatory and metabolic regulatory functions, enhanced nitrilase expression may reflect a T cell-active tumor microenvironment conducive to ICI response. In contrast, in the nonresponse group, lipoic acid metabolism was enriched, serving as a key cofactor in energy metabolism and exhibiting antioxidant activity, which may indirectly modulate immune cell function and inflammatory responses by regulating oxidative stress levels.^45^ The GO enrichment analysis (Figure 3d) showed that, in the response group, terms were predominantly associated with the biological process (BP) and cellular component (CC) categories, with biological_process and Molecular_function being the most enriched terms in BP and CC, respectively. In the nonresponse group, enrichment was also mainly observed in the BP category, followed by the CC category.

Supplementary Figure 2 summarizes the cumulative attribution scores across all features at the genus level in the Ashray_2024 cohort. Notably, the *Bacteroides* genus exhibited positive contributions in both responders (IG value = 0.8068) and non-responders (IG value = 0.0049), suggesting a consistent role in model discrimination. This dual role of *Bacteroides* stem from its unique biological characteristics in the human microbiota. The *Bacteroides* genus maintains a complex and generally beneficial relationship with the host, which may underlie its involvement in both effective and impaired responses to ICIs. However, when it escapes the gut environment, it can cause significant pathology, including bacteremia and abscess formation at multiple body sites.^46^^,^^47^ A similar pattern was observed in the Lee_2022 cohort (Supplementary Figure 3), and the *Fusicatenibacter* genus exhibited positive contributions in both responders (IG value = 0.0083) and non-responders (IG value = 0.0027). Notably, the Ashray_2024 and Lee_2022 cohorts represent different cancer types, and the highest positively contributing genera differed between the two cohorts. This variation may be associated with cancer type-specific host–microbiota interactions, highlighting the context-dependent role of gut microbes in modulating immunotherapy responses. The same attribution analysis was conducted in the Lee_2022, Maston_2018, and Fankel_2017 cohorts, with the results presented in Supplementary Figures 3–5.

Our results demonstrate that RPKM features consistently outperform taxonomic profiles in predicting ICI response. Furthermore, feature attribution analysis identified key microbial contributors associated with ICI response, suggesting that RPKM features not only enhance predictive accuracy but also reflect biologically informative signatures that are consistent with host‒microbiome interactions in immunotherapy.

### Treatment regimen heterogeneity limits cross-cohort generalizability of ICI response prediction

Given the heterogeneity in tumor types and ICI regimens, we were particularly interested in differentially evaluating the performance of RPKM features in specific cancer and ICI regimen cohorts. First, we conducted a cross-cohort evaluation across four independent cohorts, in which each cohort was designated the training set, and the models were evaluated in the remaining three cohorts to assess the generalizability of our model cross cohorts. Second, to further investigate whether the predictive performance is ICI regimen-specific, we stratified the Frankel_2017 cohort into a subset of CICB and a monotherapy subset (anti-PD-1 or anti-CTLA-4). Predictive models trained on the remaining three cohorts were subsequently evaluated on the two subsets.

**Figure 4.**
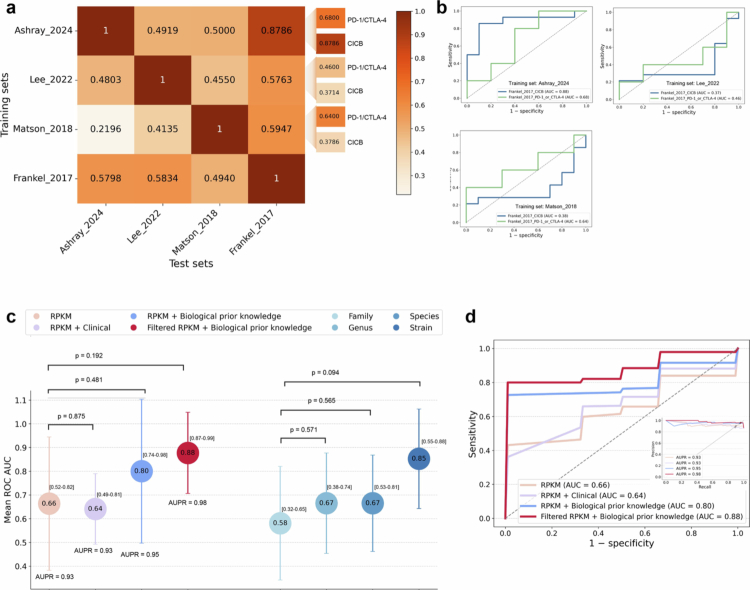
Cross-cohort performance of the BioP-VAE models. (a) Heatmap denoting the AUC scores for models using filtered RPKM features with biological prior knowledge for ICI response prediction, trained on one cohort (columns) and tested on other cohorts (rows). The rightmost columns show the AUC scores on two subsets (as test sets) derived from the Frankel_2017 cohort. The subsets are split by ICI regimen (CICB or anti-PD-1/anti-CTLA-4 monotherapy). (b) ROC curves of models trained on the Ashray_2024, Lee_2022, and Matson_2018 cohorts and tested on the subsets of Frankel_2017 for ICI response prediction. (c) Predictive performance of 12-month PFS across various combinations of RPKM features, taxonomic abundance, and selection strategies in the Ashray_2024 cohort. The circles indicate the mean AUC scores, with error bars representing the standard deviation. The AUPR values are also shown. The statistical significance between AUC scores within identical feature categories is indicated by p-values. The bracketed values denote 95% bootstrap confidence intervals (1000 replicates). (d) ROC curves corresponding to the AUC results in (c), with the inset showing the corresponding PR curves.

Figure 4a and b illustrate the cross-cohort generalization performance of ICI response prediction models based on filtered RPKM features with biological prior knowledge. The cross-cohort performance based on all RPKM features and all RPKM with biological prior knowledge is also shown in Supplementary Figure 6a and b, respectively. In Figure 4a, each cell of the heat map represents the AUC score obtained when the model was trained on one cohort and tested on another. The rightmost columns show the AUC score on two subsets (as the test set) derived from the Frankel_2017 cohort. We observed substantial variation in generalizability across different ICI regimens. The models trained on the Ashray_2024 cohort (all patients with the CICB regimen) performed well when tested on the mixed-regimen Frankel_2017 cohort (AUC = 0.8786), but the models trained on the monotherapy cohorts (Lee_2022 and Matson_2018) showed poor generalizability, with AUC scores not exceeding 0.60. Next, we divided the Frankel_2017 cohort into CICB and monotherapy subsets. Notably, the model trained on Ashray_2024 achieved a high AUC score of 0.8786 for the CICB subset, an AUC score of only 0.68 for the monotherapy subset. In contrast, models trained on the other two cohorts yielded AUC scores less than 0.64 for both subsets. The corresponding ROC curves of tests on the subsets are shown in Figure 4b.

We observed consistently poor cross-cohort predictive performance among the Lee_2022, Matson_2018, and Frankel_2017 cohorts, all of which consisted of melanoma patients. The AUC scores were mostly less than 0.60, with a maximum of 0.59 (Figure 4a), and the corresponding ROC curves are provided in Supplementary Figure 7. Furthermore, the performance on the Frankel_2017 monotherapy subset showed a slight improvement, with AUC scores reaching 0.64. In contrast, the performance on the Frankel_2017 CICB subset was notably worse, with AUC scores ranging from 0.3714 to 0.3786 (Figure 4b shows the ROC curves). Although melanoma is generally considered responsive to ICB, the underlying cohort differences still impacted the generalizability of the BioP-VAE model.

In summary, cross-cohort validation revealed that treatment regimen heterogeneity is a key factor influencing patients' ICI response. While tumor type is an important variable in determining the ICI response, it is not the sole limiting factor. While the gut microbiome remains a promising candidate for a universal biomarker, the consistently suboptimal cross-cohort performance observed in melanoma studies underscores the inherent heterogeneity of microbial signatures. This emphasizes a pressing need for larger-scale datasets to train more robust models, thereby enhancing BioP-VAE generalizability across diverse patient populations.

### Improved 12-month PFS prediction with gene-level microbial abundance features

PFS is widely used as an endpoint to evaluate the long-term clinical benefit of ICB, particularly in cancer types characterized by slow progression or heterogeneous treatment responses. Given our earlier findings that ICI therapy regimen heterogeneity influences the generalizability of our BioP-VAE model, we next aimed to determine whether RPKM features could improve 12-month PFS prediction performance in a CICB regimen of the Ashray_2024 cohort, which has complete follow-up data.

Consistent with our previous observations in ICI response prediction tasks, simply concatenating clinical features with RPKM features did not improve model performance, with the average AUC decreasing from 0.66 to 0.64 (*p* = 0.875, Figure 4c). In contrast, when applying the MI selection strategy to the RPKM features and incorporating a biological prior knowledge, model discriminative performance improved substantially, achieving the highest AUC of 0.88 (Figure 4c and d). Furthermore, we compared them with taxonomic abundance features. A consistent increase in the mean AUC score was observed with higher taxonomic resolution, ranging from the family to the strain level (as shown in Figure 4c). The strain-level abundance features provided the best predictive performance (AUC = 0.85), significantly outperforming the more common species-level abundances (AUC = 0.67). These results suggest that strain-level abundance features are more valuable in predicting landmark PFS than higher taxonomic aggregations, as finer taxonomic granularity preserves discriminative variation that is obscured at broader levels.^21^

Overall, these findings underscore the predictive advantage of RPKM features over traditional taxonomic abundance profiles, especially when integrated with biological prior knowledge. These results highlight the potential of high-resolution microbiome features to inform individualized prognosis for patients treated with ICIs.

### Divergent microbial predictors of ICI response in older and younger cohorts

The host immune system and gut microbiome are both shaped by age-related physiological changes, which may influence the ICI response of patients.^13^^,^^48^^,^^49^ We integrated the samples from Ashray_2024, Lee_2022, and Frankel_2017 cohorts and stratified the samples into two subgroups: older (>60 y) and younger (≤60 y) cohorts. Within each subgroup, we trained our ICI response predictive models using various RPKM feature sets. To further characterize group-specific microbial signatures, we performed attribution analyses using the filtered RPKM features with biological prior knowledge. The sample sizes and responder/non-responder distributions for each group are summarized in Table 1.

**Table 1.** Distribution of ICI responders and non-responders across age subgroups. Samples in each cohort were stratified into young (≤60 y) and old (>60 y) groups, and the number of responders and non-responders was summarized within each age subgroup.

Cohort	Study					
	Older (>60)			Younger (≤60)		
	Responders	Non-responders	Total	Responders	Non-responders	Total
Ashray_2024	11	42	53	15	38	53
Lee_2022	34	61	95	28	39	67
Frankel_2017	11	16	27	8	4	12
All	56	119	175	51	81	132

**Figure 5.**
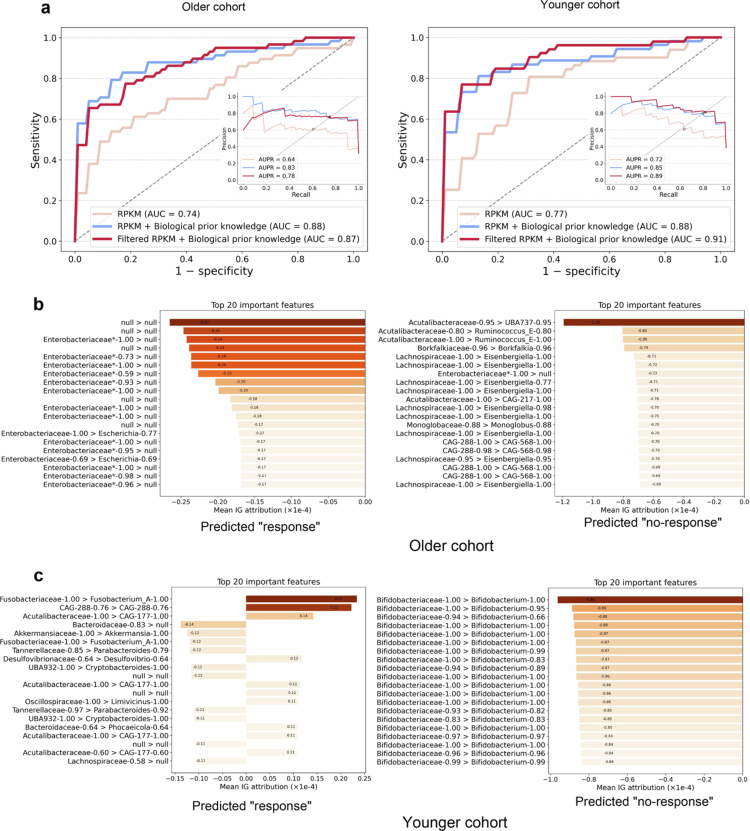
Age-stratified predictive performance and key microbial features associated with ICI response. (a) ROC curves of three RPKM feature sets in the younger and older cohorts, with the inset showing the corresponding PR curves. (b and c) Top 20 features ranked by mean IG scores, shown separately for patients predicted as responders and non-responders in the older and younger cohorts. The y-axis labels represent features in the format “FamilyName-confidence > GenusName-confidence” (e.g., “Enterobacteriaceae*-1.00 > *Escherichia*-0.77”), with the corresponding IG scores displayed on the x-axis. The absence of family or genus annotations (or both) in certain cases indicates that the corresponding contig could not be taxonomically classified based on the GTDB, and such cases are denoted as “null”.

First, the average AUCs in the older and younger cohorts were 0.74 and 0.77 using all RPKM features, respectively. Furthermore, we incorporated biological prior knowledge and found that the model achieved higher mean AUC scores of 0.88 in both the older and younger cohorts (Figure 5a). Subsequently, we evaluated the filtered RPKM features with biological prior knowledge. The model achieved an average AUC of 0.87 in the older cohort, which was slightly lower than that of the model using all RPKM features combined with biological prior knowledge. Interestingly, we observed a significant improvement in the mean AUC of the model in the young cohort, with an increase of 3%. Overall, the AUC scores based on RPKM features were consistently higher in the young cohort compared to the older cohort, suggesting that these features may have more stable predictive performance in younger patients.

Subsequently, we performed IG attribution analysis of the model trained on filtered RPKM features with biological prior knowledge. We show the top 20 contributing microbial features from each age group (Figure 5b and c) and the cumulative IG contribution aggregated at the genus level (Supplementary Figures 8 and 9). Notably, the high-attribution microbial taxa exhibited substantial differences between the younger and older cohorts (Figure 5b and c), suggesting age-specific patterns in how the microbiome relates to ICI response.

In ICI responders, the top 20 features of the older cohort were predominantly comprised of taxa belonging to the Enterobacteriaceae family. These taxa all exhibited negative IG values (Figure 5b, predicted “response”), indicating that higher abundance of these taxa was associated with a lower likelihood of ICI response. As shown in Supplementary Figure 8, the *Bifidobacterium* genus exhibits the highest positive cumulative IG value among all the genera, with a score of 0.0024, indicating that an increased abundance of this genus contributed positively to the model's prediction capability. This finding is supported by previous studies showing that the *Bifidobacterium* genus can selectively accumulate in tumors after entering the bloodstream, likely due to the hypoxic and immunosuppressive tumor microenvironment, which facilitates bacterial colonization and proliferation.^50^ Furthermore, the *Bifidobacterium* genus has been recognized as a promising delivery vehicle for tumor-specific transport^51^^,^^52^ and has been shown to increase antitumor immune surveillance by increasing the abundance of tumor-infiltrating $\mathrm{CD}8^{+}$ T cells.^50^ Instead, the *Faecalibacterium* genus shows the largest negative cumulative IG value (IG value = −0.0043), suggesting that its increased abundance may exert a suppressive effect on the model's predictive outcome. The younger cohort exhibited a more diverse set of high-contributing taxa, including the Fusobacteriaceae, CAG-288, and Acutalibacteraceae families (Figure 5c, predicted “response”). These features also had positive IG values, suggesting a supportive role in ICI efficacy within this age cohort. The broader diversity of predictive microbial taxa in younger individuals may reflect a more complex interplay between the microbiome and host immunity. As shown in Supplementary Figure 9, the *UBA11524* genus exhibits the highest positive cumulative IG value among all the genera (IG value = 0.0027). Although the *UBA11524* genus is an uncultured or poorly characterized candidate genus, its strong positive feature importance suggests a potentially significant role in ICI response studies.

In non-responders, the top 20 features in the older cohort all showed negative IG values and were primarily comprised of the Acutalibacteraceae and Lachnospiraceae families (Figure 5b, predicted “no-response”). In Supplementary Figure 8, the *Collinsella* genus exhibits the highest positive cumulative IG value (0.0016) among all the genera in the predicted no-response samples from the elderly group, followed by the *CAG* (0.0009) genus. These findings suggest that the elevated abundance of these taxa may be associated with the no-response phenotype in older patients. Previous studies have associated the *Collinsella* genus with metabolic dysregulation, inflammatory conditions, and impaired gut barrier function, which may indirectly modulate the immune system and consequently affect responses to immunotherapy.^53^^,^^54^ All the top 20 features in non-responders from the younger cohort belonged to the Bifidobacteriaceae family, with consistently negative IG values (Figure 5c, predicted “no-response”). This finding indicates that increased abundance of these taxa was associated with a reduced model tendency to predict no-response, implying a potential positive association with ICI response. In Supplementary Figure 9, the *CAG* genus exhibited the highest positive cumulative IG value (0.0014) among all the genera in the predicted no-response samples of the young cohort, followed by *Lahnospira* (0.0012). Interestingly, we observed that the *CAG* genus exhibited bidirectional importance in our ICI prediction model within the young cohort, suggesting a complex and multifaceted biological role that may contribute to both the promotion and inhibition of ICI responses.

These results indicate that RPKM features can effectively predict ICI responses in both age subgroups. Through IG attribution analysis, we further identified potential microbial biomarkers related to the ICI response and revealed age-specific differences in the microbiota associated with immunotherapy outcomes. Additionally, we conducted additional stratified analyses across clinically relevant subgroups (Supplementary Figures 13–16, and Supplementary Table 2). The genes remained stable in patients without antibiotic exposure, in leave-one cohort-out validation, across different treatment regimens, and across cancer types.

## Discussion

In this study, we developed a supervised deep learning model named BioP-VAE, which integrates protein sequence embeddings from reference genomes as biological prior knowledge and leverages gene-level microbial abundance as input features. We systematically evaluated its predictive performance for clinical response and 12-month PFS in ICI therapy. This approach expands the utility of microbial features in immunotherapy prediction and provides a new foundation for developing more generalizable and non-invasive biomarkers.

Our results demonstrated that gene-level microbial abundance features outperformed traditional taxonomy-based features across family, genus, species, and strain levels, achieving an AUC score of 0.89. These findings highlight the importance of capturing microbial functional activity at the gene-content level. Microbial functions are generally more conserved than taxonomic composition, reflecting substantial functional redundancy across taxa.^55^ Moreover, species-level profiles may obscure extensive strain-level genomic variation, whereas gene-level features provide a more direct representation of microbial functional capacity relevant to host‒microbiome interactions.^56^ This functional redundancy is biologically plausible, as homologous genes from phylogenetically distinct microorganisms can encode similar biochemical activities. Consequently, different taxa may contribute to comparable alterations in gut microbial function that are associated with specific pathological processes.^57^

Several recent studies have explored gut microbiome signatures associated with ICI response using species- or strain-level abundance profiles. For example, Thomas et al. defined cross-cancer Gut OncoMicrobiome Signatures based on species-level data,^58^ whereas Gunjur et al. reported improved predictive performance using strain-level signatures,^21^ particularly when treatment regimens were matched between training and testing cohorts. In contrast, our study adopts gene-level features and integrates protein-level biological prior knowledge, enabling representation beyond taxonomic assignment. Notably, gene-level features have also demonstrated superior cross-cohort robustness in multi-cohort studies of Crohn's disease,^59^ supporting the concept that gene-level features generalize more than taxonomy. Our results extend this principle to ICI response prediction under cross-cohort validation settings.

Attribution analysis further identified key microbial taxa associated with ICB response in the CICB samples, with the Rikenellaceae, Ruminococcaceae, and Lachnospiraceae families contributing positively in responders, while the Desulfovibrionaceae and Bacteroidaceae families were enriched in non-responders. Moreover, we found that heterogeneity in ICB treatment strategies significantly influenced model generalizability across cohorts. Notably, the CICB therapy exhibited more stable predictive markers in cross-cancer, cross-cohort analyses. In the Ashray_2024 cohort with comprehensive clinical follow-up, gene-level abundance features substantially enhanced the predictive performance for 12-month PFS (AUC = 0.88). Age-stratified analyses revealed distinct microbial predictors of ICI response, with the *Bifidobacterium* genus showing higher attribution scores in older responders and *UBA11524* contributing more strongly in younger responders, indicating potential age-associated differences in microbial contribution patterns.

This study has several limitations that should be addressed in future work. First, although the overall sample size in this study is relatively large, it remains limited and may restrict the generalizability of our predictive model. The technical variations in fecal collection and DNA extraction protocols across cohorts may introduce batch effects, potentially affecting microbial profiling and downstream analyses. Second, our custom reference genome set includes samples primarily from patients treated with CICB regimens. Expanding this set to incorporate samples from a broader spectrum of treatment strategies could further enhance the robustness and translational potential of the model. Additionally, our study relies on bacterial reference gene catalogs and does not explicitly capture contributions from non-bacterial microbial kingdoms, such as fungi or viruses. Recent multi-cohort analyses have demonstrated that trans-kingdom gut microbiota, including both bacterial and eukaryotic species, are associated with ICI response across multiple cancer types and can serve as predictive biomarkers.^60^ Incorporating fungal and viral gene features through expanded reference gene catalogs may therefore help capture additional microbial signals and improve predictive performance in future studies. Finally, this study focused primarily on gene-level features of the gut microbiome and did not incorporate host-related multidimensional biological information, such as genetic variation, tumor immune microenvironment characteristics, and metabolite profiles. Given that the efficacy of ICI is influenced by a complex interplay of host factors, the omission of this key information may limit the model's ability to fully account for host contributions to the treatment response. Looking forward, the BioP-VAE framework could be applied in prospective, multi-center clinical trials to address these limitations. In such a design, patients are recruited prior to treatment, with longitudinal stool samples collected alongside clinical annotations and multi-omics host data. By integrating objective clinical endpoints – such as response or survival – the model could be iteratively refined to incorporate this additional biological information.

We anticipate that this work provides a number of readily implementable insights to help future microbiome-based immunotherapy research. Given the substantial cost and potential toxicity associated with immune checkpoint inhibitors, improved prediction of treatment response may support more effective patient stratification and help avoid ineffective therapies. First, our findings highlight the predictive value of gene-level microbial abundance features in ICI response. This feature may better reflect microbial functional capacity, providing a more informative readout for biomarker discovery. Second, our supervised deep learning model, which incorporates biological prior knowledge, demonstrates the potential to identify broadly applicable microbial signatures, laying the groundwork for the future development of multi-cancer microbiome-derived diagnostics or therapeutic adjuncts. Finally, we observed that microbial signatures associated with CICB regimens may be generalizable across cancer types and geographic regions. Future studies should differentiate these signatures from those related to monotherapy regimens to enhance the precision of personalized clinical applications.

## Methods

### Data acquisition

We used PubMed to search for studies that published fecal shotgun metagenomic data from patients receiving ICB therapy. Raw FASTQ files of 347 fecal samples from four studies were downloaded from the European Nucleotide Archive (ENA).

We included cohorts that analyzed fecal samples collected within ±15 d of ICB initiation, performed Illumina paired-end shotgun metagenomic sequencing, and provided metadata on either RECIST best overall response (BOR) or pathological response. A summary of the characteristics of the four cohorts is provided in Supplementary Table 1. We extracted ICB treatment response labels for the included cohorts based on annotations summarized in the study by Lin et al.^60^

In the clinical factor contribution analysis of the Ashray_2024 cohort, we included the following 15 clinical metadata variables that were potentially associated with treatment response and/or gut microbial composition: patient age (at the time of trial initiation), sex, body mass index (BMI), ECOG performance status, histology cohort (based on pathology reports), extent of measurable tumor (calculated as the sum of RECIST target lesion diameters based on the screening CT scan), study site, season of fecal sample collection, antibiotic use, proton‒pump inhibitor use, chemotherapy use, blood NLR, platelet count, albumin level, and lactate dehydrogenase (LDH) level.

### Quality control and host DNA removal of fecal shotgun sequencing data

We performed quality control on raw paired-end sequencing reads using Sickle (v1.33)^61^ in paired-end mode. Bases with quality scores below 20 were trimmed (−q 20), and reads shorter than 20 bp after trimming were discarded (default minimum length). Singleton reads generated during trimming were retained at this stage but excluded from downstream analyses, where only properly paired reads were used. Following quality filtering, a second round of host contamination removal was performed using the GRCh38^27^ human genome assembly as a reference. Cleaned reads were aligned to the reference genome using the Bowtie2^62^ tool, and alignment files were processed with the Samtools tool (v1.21)^63^ to extract unmapped reads, effectively filtering out sequences of human origin. The resulting non-human reads were then converted back to the FASTQ format to serve as input for downstream metagenomic analyses. All cohorts were processed using an identical preprocessing pipeline to ensure comparability.

### Reference genome acquisition and annotation

In this study, we constructed a custom reference genome database using non-human paired-end sequencing reads from the Ashray_2024 cohort (see the black arrows in Figure 1 for the workflow). Specifically, SPAdes (v4.00)^64^ was employed to perform individual de novo assemblies for each sample using the option “–meta,” which is optimized for metagenomic data. Following assembly, the contigs from all the samples were merged into a unified dataset. To ensure reference quality, short contigs (<​​​​​500 bp) were removed. Then, we applied MetaBAT2^65^ with default parameters (option –minContig 1500) to perform genome-level binning on the merged contigs. MetaBAT2 was applied based on sequence composition features without incorporating contig coverage profiles, as the objective of this step was to cluster contigs into coarse genome-level partitions for reference organization rather than to reconstruct high-quality metagenome-assembled genomes. For each bin, the contigs were ranked by length, and two non-redundant microbial reference genome sets were generated. The first reference genome set included the longest contig from each bin and was used for constructing protein-level biological prior knowledge, consisting of 1269 contigs in total. The second reference genome set included up to the top 200 contigs from each bin (using all available contigs if fewer than 200 were present) and was used as the reference database for estimating RPKM features, consisting of 161,107 contigs in total. This design choice was based on the observed distribution of contig counts: although a few bins contained tens of thousands of contigs, the majority were relatively small (median = 142 contigs), with 58.6% of bins containing fewer than 200 contigs (Supplementary Figure 10a). Mapping rate statistics across the four cohorts (Supplementary Figure 10b) further confirmed the robustness of this strategy.

To interpret the output of the model in this study, we performed taxonomic annotation of the constructed reference genomes (top 200 contigs) using the CAT software (v5.2.3).^66^ The CAT pipeline relies on two essential components: Prodigal (v2.6.3)^67^ for predicting open reading frames (ORFs) and DIAMOND (v2.1.11)^68^ for high-speed protein sequence alignment. Taxonomic classification was carried out following the default workflow recommended by CAT. The annotation was performed using GTDB,^39^ version dated November 20, 2023. Based on the alignment results, CAT assigned taxonomic labels to each contig using a lowest common ancestor (LCA) algorithm, thereby enabling the identification of microbial lineages at multiple taxonomic levels (e.g., phylum, class, genus, and species). The final output consisted of a tabular classification report for all input contigs.

Functional enrichment analysis was performed on the top 20 contigs ranked by IG contribution scores. Gene prediction was carried out using Prodigal (v2.6.3)^67^ in metagenomic mode to obtain the predicted protein sequences. These sequences were subsequently compared against the eggNOG database to retrieve comprehensive functional annotations.^69^ We used the functional annotations of the reference genome (top 200 contigs) as the background set to perform KEGG and GO enrichment analyses. For visualization, the top 10 KEGG pathways and the top 5 GO terms in each of the three categories were selected, and we used bubble plots and bar charts to display gene counts and significance levels for each pathway and term.

### Computation of RPKM features and construction of protein-level biological prior knowledge

After constructing the reference genomes (top 200), we quantified RPKM features based on read mapping using CoverM (v0.7.0).^70^ For each sample, quality-filtered and human-decontaminated paired-end reads were aligned to the custom reference genome using the CoverM contig module, which internally utilized Minimap2^71^ as the default aligner. CoverM was run with default parameters. The RPKM mode (option -m rpkm) was selected to produce abundance values normalized by both contig length (in kilobases) and total mapped reads (in millions), thereby accounting for differences in contig size and sequencing depth. The paired-end reads were provided using the options −1 and −2, and eight threads were used for parallel processing (option -t 8). Output files were written in tab-delimited format, reporting RPKM values for each contig across samples. The workflow is illustrated by the green arrows in Figure 1. With n samples and M contigs (M = 161,107), the gene-level microbial abundance output (RPKM features) was a matrix (n×M).

To obtain protein-level biological prior knowledge representations, we first predicted protein sequences from the constructed reference genome (top 1 contig) using Prodigal (v2.6.3).^67^ Prodigal was executed in metagenomic mode (option -*p* meta), and the output files included gene feature annotations in the GFF format (option -o) and the translated amino acid sequences in the FASTA format (option -a). As a result, we obtained a total of P (P=68,160) predicted protein sequences across all the input contigs. Subsequently, each protein sequence was embedded into a fixed-length vector representation using a pretrained ESMFold^72^ model developed by the Meta Fundamental AI Research Protein Team (FAIR). ESMFold is a transformer-based protein language model trained on millions of diverse protein sequences using self-supervised learning that is capable of capturing structural and functional characteristics directly from amino acid sequences. In this study, the ESMFold (esm2_8M_UR50D) model was used to generate a 320-dimensional embedding for each predicted protein sequence, resulting in a final protein-level prior knowledge matrix of size P×320.

### Architecture of the designed BioP-VAE model

We propose the BioP-VAE model based on the VAE^26^ framework. The BioP-VAE model comprises three components: a Prior encoder that integrates biological prior knowledge to shape the RPKM features of the latent space; a decoder that learns RPKM latent representations from the output of the prior encoder, and a classifier that performs downstream classification tasks using the prior-informed latent features. The overall architecture of BioP-VAE is illustrated in Supplementary Figure 1. Each input sample is represented as a vector $x\in R^{M}$, where M denotes the dimensionality of RPKM features. A batch of samples forms a matrix $X\in R^{B\times M}$ for batch size B. Additionally, we utilize a fixed biological prior knowledge matrix $R\in R^{P\times 320}$, which encodes protein-level features. These reference features are not updated during training.

The prior encoder maps x through two fully connected layers: the first fully connected layer with batch normalization: $R^{B\times M}\to R^{B\times 512}$, and the second layer: $R^{B\times 512}\to R^{B\times 256}$. This yields the mean vector $\mu \in R^{B\times 256}$, which serves as the mean vector for reparameterized sampling of the latent variable $z\in R^{B\times 256}$:

z=μ+ϵ,ϵ∼N(0,I).

The biological prior knowledge matrix $R\in R^{P\times 320}$ is passed through a fully connected layer to project it into the latent space dimension $R\prime \in R^{P\times 256}$. This projected biological prior knowledge tensor is expanded along the batch dimension to $R^{P\times B\times 256}$, and the samplewise latent mean $\mu \in R^{B\times d}$ (d=256) is reshaped to $R^{1\times B\times d}$ to serve as the query in multi-head attention. Both the key and value tensors are set to the expanded R′. The multi-head attention output is layer-normalized to produce the final biological-aware embedding $a\in R^{B\times d}$:

$$a=\mathrm{LayerNorm}\left({\mathrm{Concat}}_{h=1}^{H}[\mathrm{softmax}\left(\frac{\mu W_{q}^{h}{\left(R\prime W_{k}^{h}\right)}^{⊤}}{\sqrt{d_{h}}}\right)R\prime W_{v}^{h}]\right)W_{o}.$$

For each attention head, $W_{q}^{h}$, $W_{k}^{h}$, and $W_{v}^{h}\in R^{d\times d_{h}}$ are the learnable weights for the query, key, and value, respectively, where $d_{h}$ is the dimension of each head. Each head computes attention weights by applying a scaled dot-product between the transformed query and key vectors, followed by a softmax operation. The outputs from all H attention heads are concatenated and passed through a shared output matrix $W_{o}\in R^{d\times d}$. The final attended representation $a\in R^{B\times d}$ is obtained by applying a layer normalization operation to the concatenation. This module enables the model to integrate biological prior knowledge into the latent representation of each sample in a context-aware manner.

To reconstruct the RPKM features, the decoder module fuses the latent vector $z\in R^{B\times 256}$ with biological-aware embedding $a\in R^{B\times d}$ using a weighted sum $\tilde{z}=\lambda z+(1-\lambda )a$, with λ=0.7. The fused representation $\tilde{z}\in R^{B\times 512}$ is then passed through two fully connected layers: the first maps it to $R^{B\times 512}$ and applies batch normalization and ReLU activation, and the second projects it to the original input dimension $R^{B\times M}$ with batch normalization. A final sigmoid activation yields the reconstructed RPKM features $x\prime \in R^{B\times M}$.

The classifier module predicts binary labels from the latent vector $\mu \in R^{B\times 256}$. It comprises two hidden layers with fully connected layers, each followed by batch normalization and ReLU activation, reducing the dimensions to $R^{B\times 128}$ and then $R^{B\times 64}$. A final fully connected layer with sigmoid activation outputs the predicted probability $\hat{y}\in R^{B}$ for each sample.

### Loss function

The BioP-VAE model is optimized using a composite loss function that integrates reconstruction fidelity, latent prior regularization, and classification accuracy. The total loss is defined as follows:

$$L_{\mathrm{total}}=\lambda_{\mathrm{rec}}L_{\mathrm{rec}}+\lambda_{\mathrm{KL}}L_{\mathrm{KL}}+L_{\mathrm{cls}.}$$

$L_{\mathrm{rec}}$ is the reconstruction loss, which is computed as the mean squared error (MSE) between the reconstructed RPKM features $x\prime \in R^{B\times M}$ and the true RPKM feature $x\in R^{B\times M}$:

$$L_{\mathrm{rec}}=\frac{1}{B}\sum_{i=1}^{B}‍∥x\prime_{i}-x_{i}{∥}^{2}.$$

i indexes individual samples in a batch of size B. $L_{\mathrm{KL}}$ is the Kullback‒Leibler (KL) divergence between the approximate posterior and a standard Gaussian prior. Given the latent mean vector $\mu \in R^{B\times d}$ and assuming unit variance, the KL loss is given by:

$$L_{\mathrm{KL}}=\frac{1}{2}\sum_{j=1}^{d}‍\mu_{j}^{2},$$

where d denotes the dimensionality of the latent space and $\mu_{j}$ is the mean of the j-th latent dimension, with j=1,2,…,d. $L_{\mathrm{cls}}$ is the binary cross-entropy loss between the predicted probabilities $\hat{y}\in R^{B}$ and the truth labels $y\in \{0,1{\}}^{B}$:

$$L_{\mathrm{cls}}=-\sum_{i=1}^{B}‍\left[y_{i}\;\log\;{\hat{y}}_{i}+(1-y_{i})\log(1-{\hat{y}}_{i})\right].$$

The scalar weights $\lambda_{\mathrm{rec}}=0.1$ and $\lambda_{\mathrm{KL}}=0.2$ are used to weight the reconstruction loss and the KL divergence, respectively, in the total loss function, thereby controlling their relative influence during model training.

### Details of training

We employed fivefold cross-validation to ensure robust model evaluation, and the results displayed in this study are the average values of the fivefolds, with each fold trained for 500 epochs using a batch size of 8. To control for randomness, the random seed was fixed at 42 for both data splitting and model initialization. Prior to modeling, sample features were aligned by using the same reference genome to ensure consistent feature dimensionality across all samples, and all features were standardized using z-score scaling. The four cohorts were processed independently at the raw sequencing stage, and gene-level abundance profiles were generated for each cohort using the same preprocessing pipeline. Integration across cohorts was subsequently conducted at the feature level rather than by merging raw sequencing data. Model parameters were optimized using the Adam optimizer,^73^ which combines adaptive learning rate estimation with momentum to accelerate convergence. The learning rate was set to $1\times 10^{-3}$. The accuracy, AUC, and AUPR were used to evaluate the performance of our model. All the models in this study were implemented using Python (v3.11.11) and PyTorch (v2.5.1, CUDA12.1). Matplotlib (v3.10.0) and Seaborn (v0.13.2) were used to create plots and figures. The training and inference processes were carried out on an NVIDIA GTX 1080Ti (24 GB).

## Supplementary Material

Supplementary Material Supplementary.pdf

## Data Availability

All raw shotgun metagenomic sequencing data are publicly available from either the respective studies or the ENA under the following accession numbers: PRJEB49516 (Ashray_2024), PRJEB43119 (Lee_2022), PRJNA399742 (Matson_2018), and PRJNA397906 (Frankel_2017). Clinical metadata were retrieved from the corresponding publications, sequencing repositories, or associated GitHub resources, as described in the Methods section.

## Code availability

All codes can be found on GitHub at https://github.com/RanSuLab/GeneGut-ICIPredict and are freely available under the permissive MIT license.
