# Supplementary material for: Gene-level gut microbiome signatures as predictive biomarkers for response to immune checkpoint inhibitors across multiple cancer types
Source: Gut Microbes. 2026 Apr 23;18(1):2662690. doi: 10.1080/19490976.2026.2662690 (PMC13114121; doi:10.1080/19490976.2026.2662690)
Supplement: Supplementary Material — Supplementary.pdf [file KGMI_A_2662690_SM8483.pdf]

CONTENTS

Supplementary Figure 1 1
Supplementary Figure 2 2
Supplementary Figure 3 3
Supplementary Figure 4 4
Supplementary Figure 5 5
Supplementary Figure 6 6
Supplementary Figure 7 7
Supplementary Figure 8 8
Supplementary Figure 9 9
Supplementary Figure 10 10
Supplementary Figure 11 11
Supplementary Figure 12 12
Supplementary Figure 13 13
Supplementary Figure 14 14
Supplementary Figure 15 15
Supplementary Figure 16 16
Supplementary Table 1 17
Supplementary Table 2 18

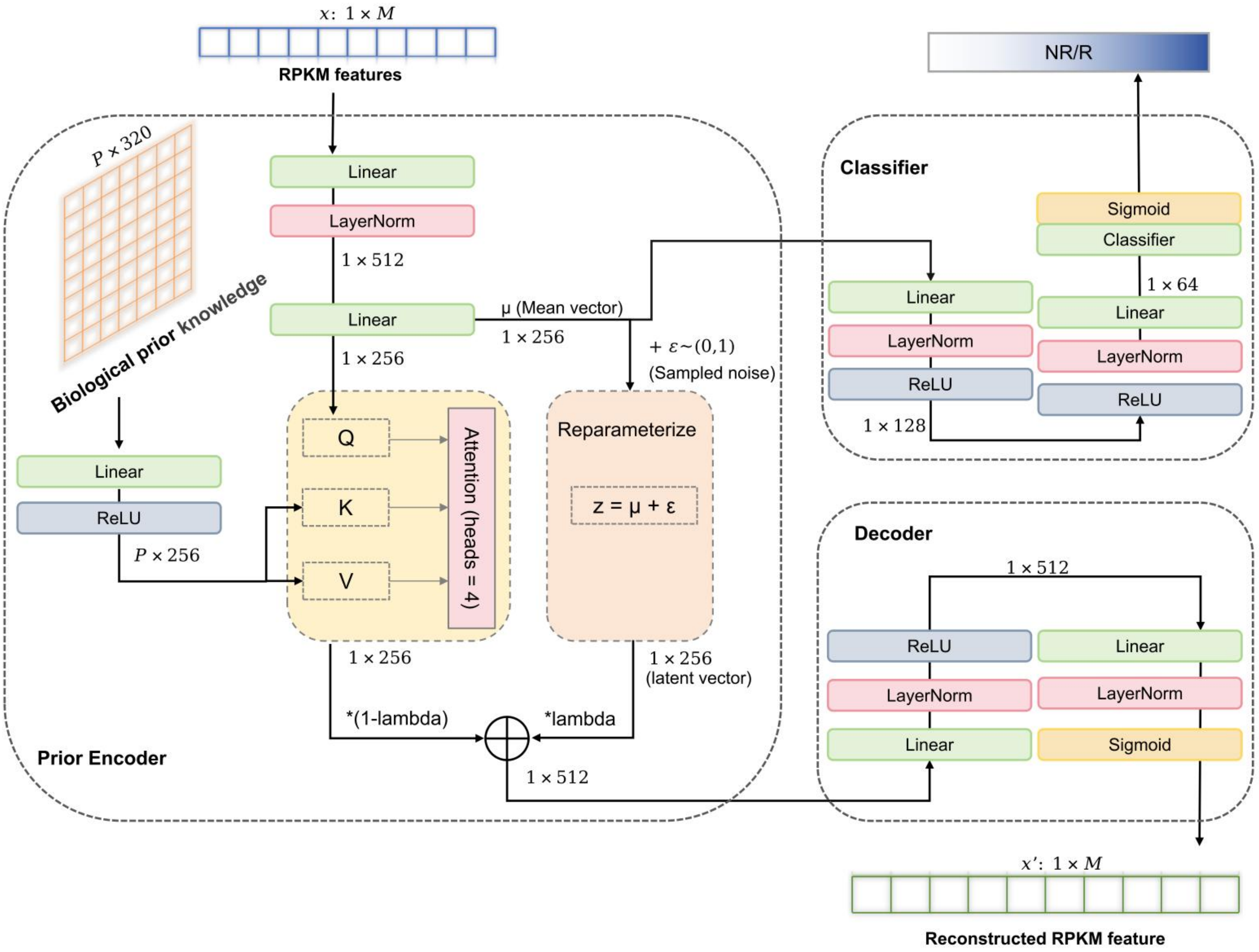

Supplementary Figure 1: Overview of the BioP-VAE architecture. The model comprises three main components: (1) Prior Encoder: Each input sample is a RPKM feature vector of size 161,107 or 80,000 (for filtered features). It is first processed through two fully connected layers that reduce the dimensionality to 256. This results in a latent mean vector of size 256. In parallel, a fixed biological prior knowledge matrix with dimensions 68,160 × 320 is projected into a 256-dimensional space. This prior is used as the key and value in a multi-head attention module, with the latent mean vector serving as the query. The output of the attention mechanism is a biological-aware embedding of size 256, which incorporates prior knowledge into the latent space. (2) Decoder: A latent vector of size 256 is sampled from the latent mean, then combined with the biological-aware embedding through a weighted sum to form a fused vector of size 256. The fused representation is passed through two fully connected layers to reconstruct the original RPKM feature vector of size 161,107. A sigmoid activation function is applied to produce the final reconstructed output. (3) Classifier: The latent mean vector of size 256 is also passed to a classifier module for downstream prediction. It is processed through two fully connected layers that reduce the dimensionality to 64, each followed by batch normalization and ReLU activation. A final fully connected layer outputs a single predicted probability for binary classification.

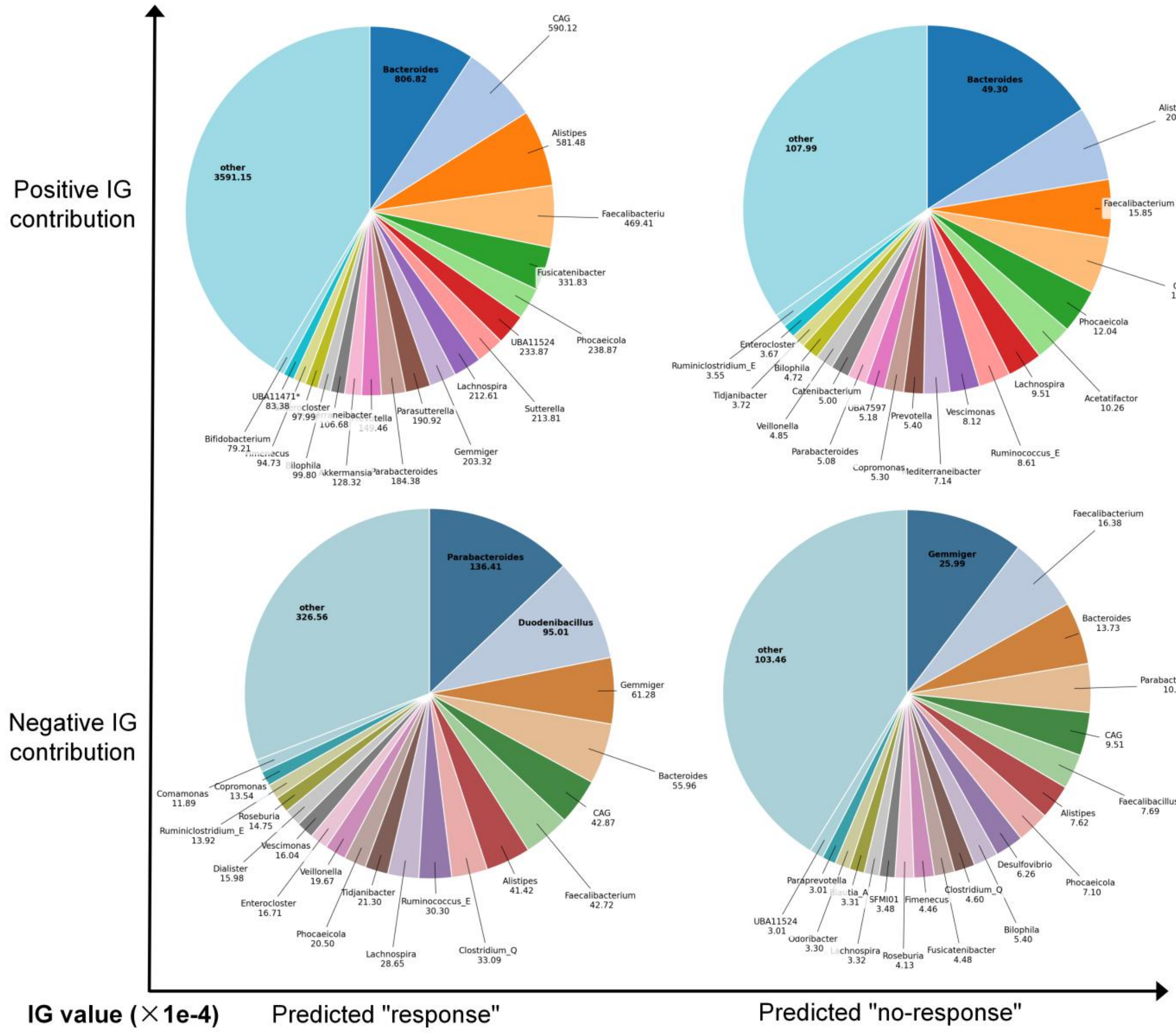

Supplementary Figure 2: The IG attribution analysis on the Ashray_2024 cohort using gene-level abundance features (filtered RPKM + biological prior knowledge). IG scores were calculated from the validation sets of five-fold cross-validation, averaging the attribution values from correctly predicted samples across all folds. The feature (contig) name/label was annotated according to the GTDB database. Pie charts showing the cumulative IG contribution aggregated at the genus level, with positive and negative IG values summed separately for responder and non-responder groups, reflecting the overall genus-level influence on ICI therapy response prediction outcomes.

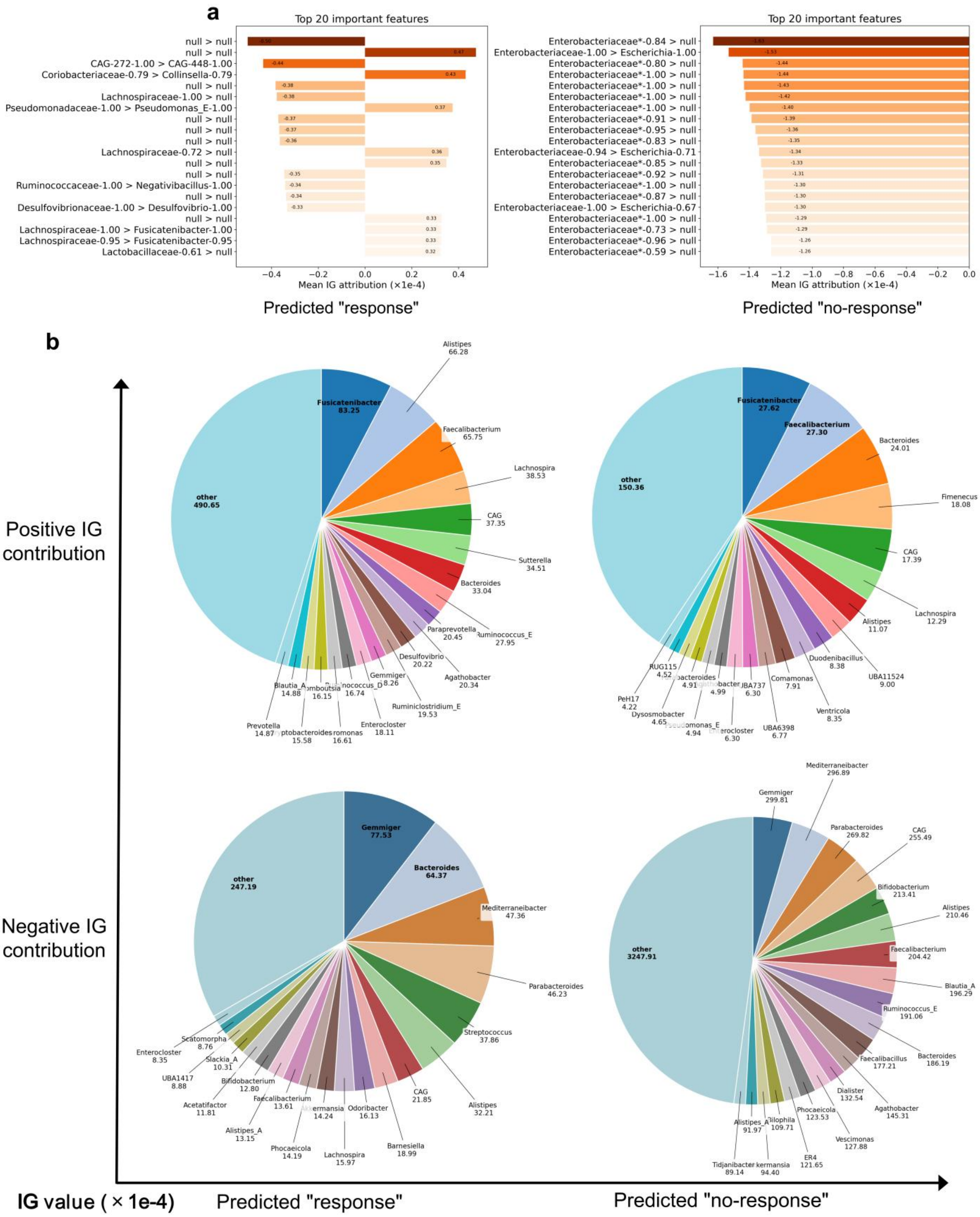

Supplementary Figure 3: The IG attribution analysis on the Lee_2022 cohort using gene-level abundance features (filtered RPKM + biological prior knowledge). IG scores were calculated from the validation sets of five-fold cross-validation, averaging the attribution values from correctly predicted samples across all folds. The feature (contig) name/label was annotated according to the GTDB database. **a,** Top 20 features ranked by the mean IG values in patients predicted as responders and non-responders. The y-axis labels represent features in the format "FamilyName-confidence > GenusName-confidence" (e.g., "Rikenellaceae-1.00 > Alistipes-1.00"), with the corresponding IG values displayed on the x-axis. The absence of family or genus annotations (or both) in certain cases indicates that the corresponding contig could not be taxonomically classified based on GTDB. **b,** Pie charts showing the cumulative IG contribution aggregated at the genus level, with positive and negative IG values summed separately for responder and non-responder groups, reflecting the overall genus-level influence on ICI therapy response prediction outcomes.

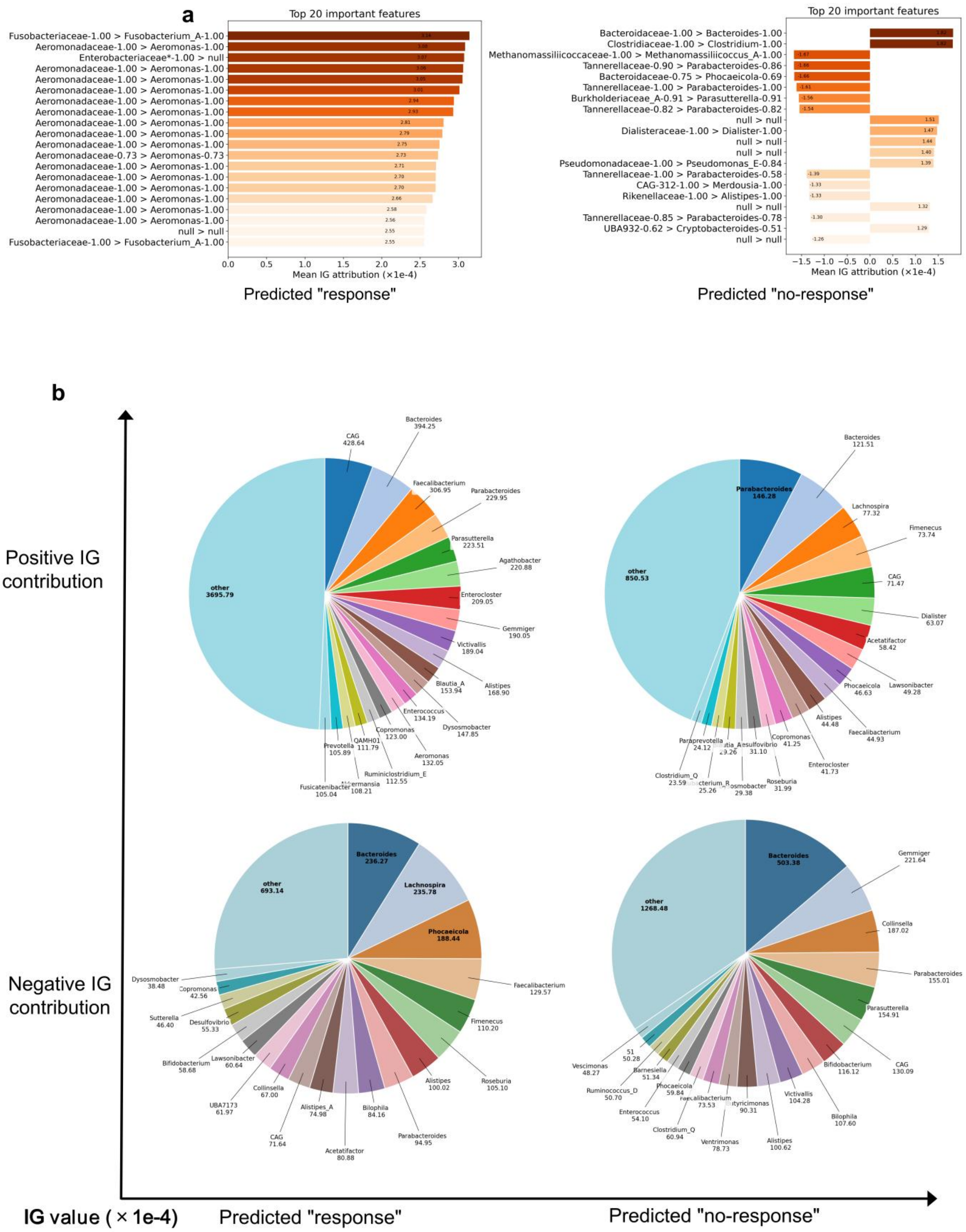

Supplementary Figure 4: The IG attribution analysis on the Matson_2018 cohort using gene-level abundance features (filtered RPKM + biological prior knowledge). IG scores were calculated from the validation sets of five-fold cross-validation, averaging the attribution values from correctly predicted samples across all folds. The feature (contig) name/label was annotated according to the GTDB database. **a,** Top 20 features ranked by the mean IG values in patients predicted as responders and non-responders. The y-axis labels represent features in the format "FamilyName-confidence > GenusName-confidence" (e.g., "Rikenellaceae-1.00 > Alistipes-1.00"), with the corresponding IG values displayed on the x-axis. The absence of family or genus annotations (or both) in certain cases indicates that the corresponding contig could not be taxonomically classified based on GTDB. **b,** Pie charts showing the cumulative IG contribution aggregated at the genus level, with positive and negative IG values summed separately for responder and non-responder groups, reflecting the overall genus-level influence on ICI therapy response prediction outcomes.

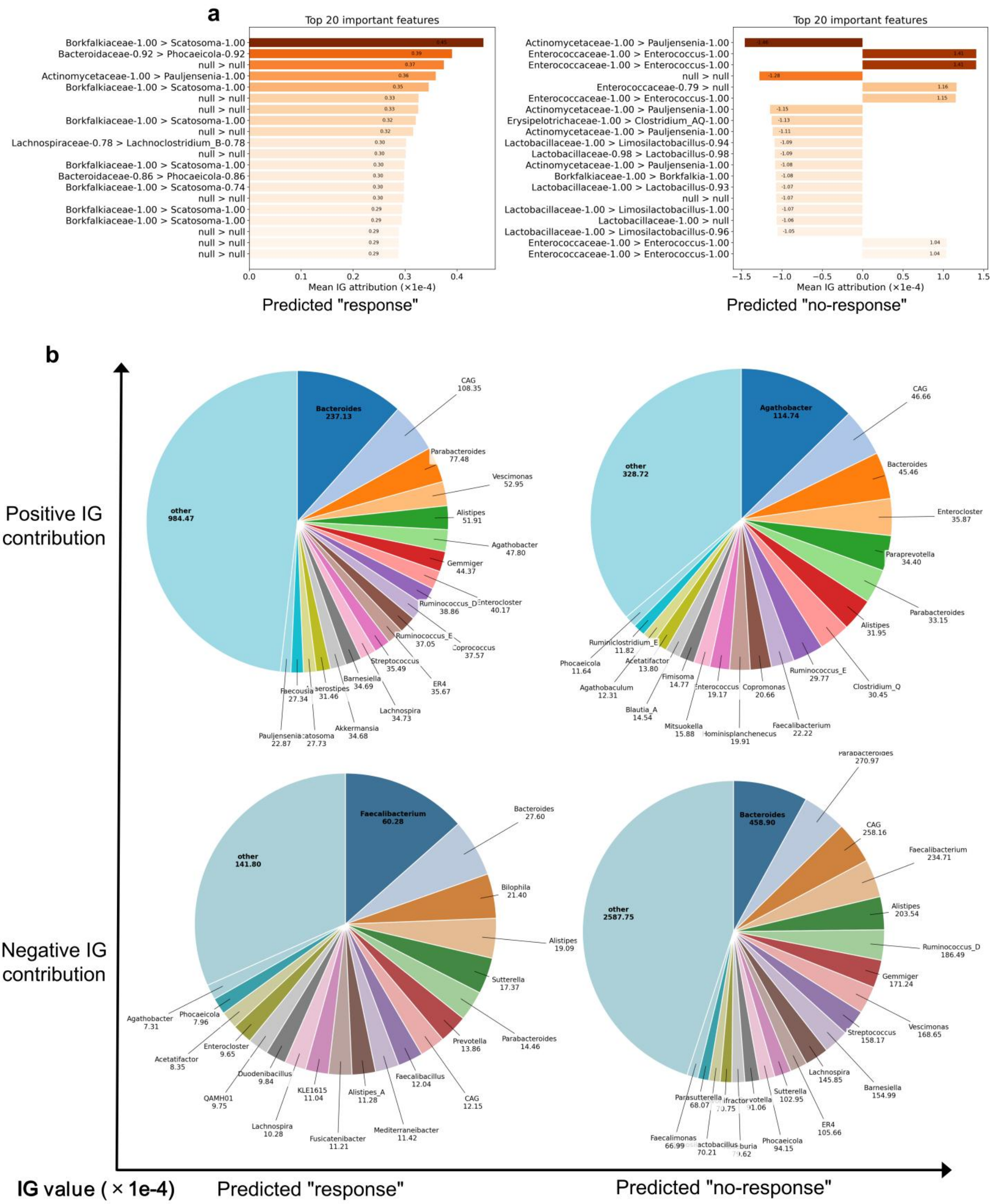

Supplementary Figure 5: The IG attribution analysis on the Frankel_2017 cohort using gene-level abundance features (filtered RPKM + biological prior knowledge). IG scores were calculated from the validation sets of five-fold cross-validation, averaging the attribution values from correctly predicted samples across all folds. The feature (contig) name/label was annotated according to the GTDB database. **a,** Top 20 features ranked by the mean IG values in patients predicted as responders and non-responders. The y-axis labels represent features in the format "FamilyName-confidence > GenusName-confidence" (e.g., "Rikenellaceae-1.00 > Alistipes-1.00"), with the corresponding IG values displayed on the x-axis. The absence of family or genus annotations (or both) in certain cases indicates that the corresponding contig could not be taxonomically classified based on GTDB. **b,** Pie charts showing the cumulative IG contribution aggregated at the genus level, with positive and negative IG values summed separately for responder and non-responder groups, reflecting the overall genus-level influence on ICI therapy response prediction outcomes.

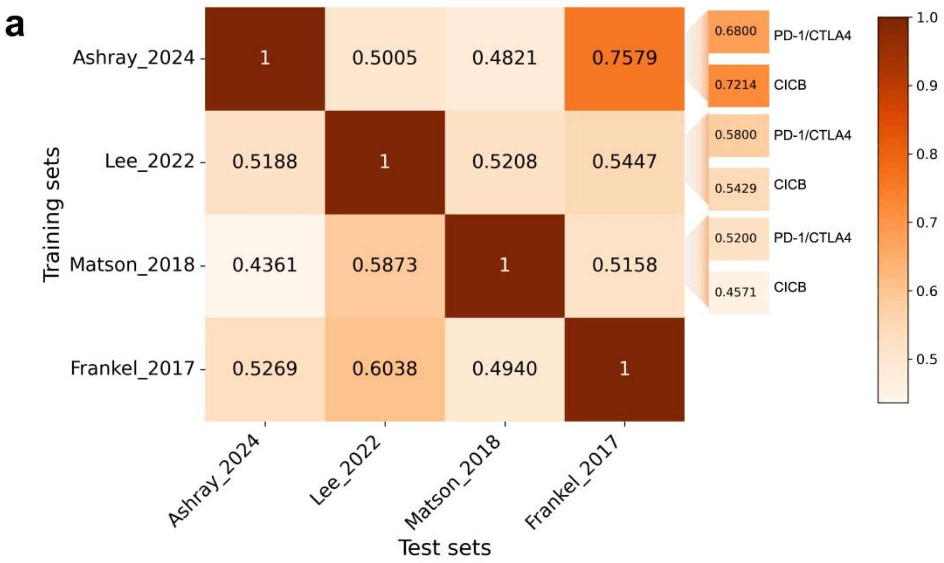

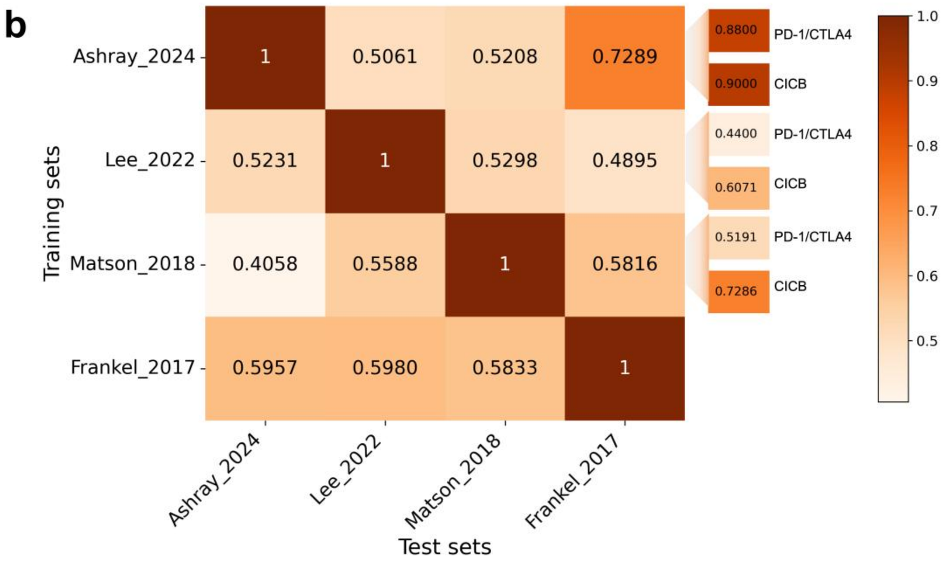

Supplementary Figure 6: a, Heatmap denoting the AUC scores for models using all RPKM features, trained on one dataset (columns) and tested on another (rows). The rightmost columns show the AUC scores on two subsets (as test sets) derived from the Frankel_2017 cohort. **b,** Heatmap denoting the AUC scores for models using all RPKM features with biological prior knowledge, trained on one dataset (columns) and tested on another (rows). The rightmost columns show the AUC scores on two subsets (as test sets) derived from the Frankel_2017 cohort. The subsets are split by ICI regimen (CICB or anti-PD-1/anti-CTLA-4 monotherapy).

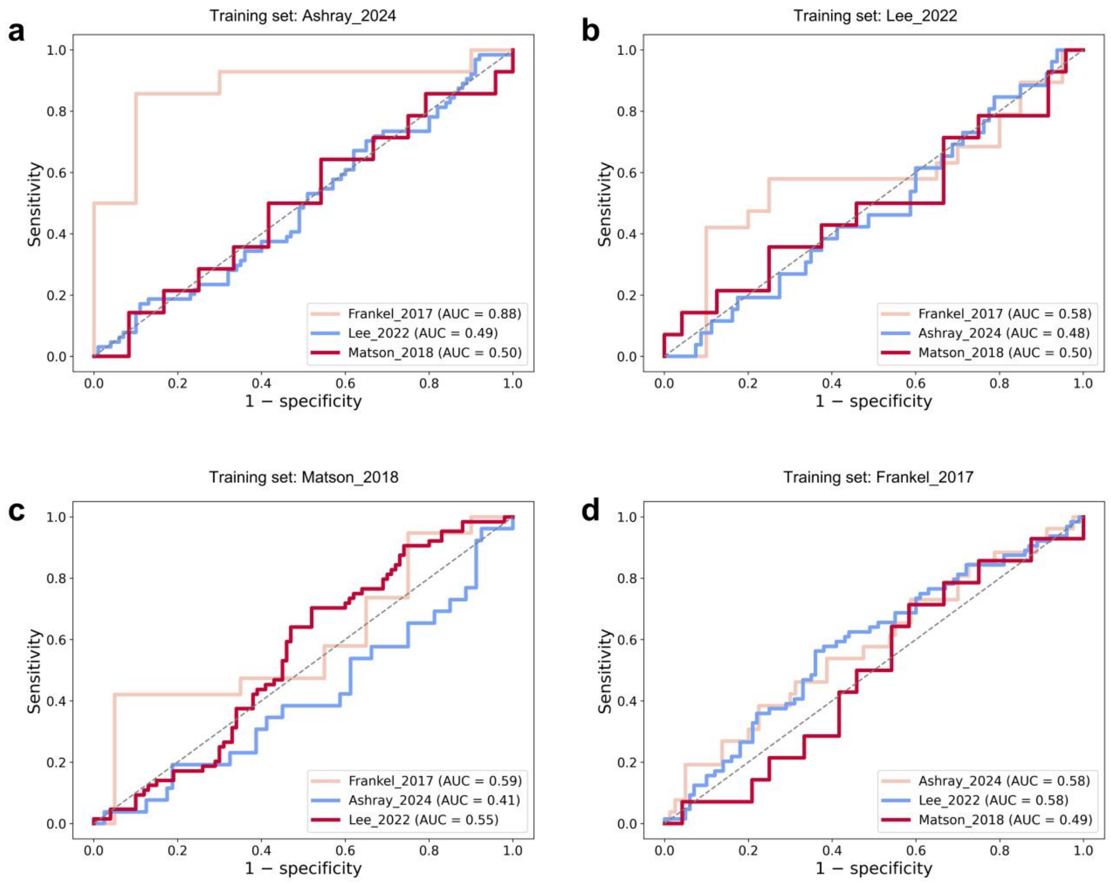

Supplementary Figure 7: ROC curves of models trained on the Ashray_2024 (**a**), Lee_2022 (**b**), Matson_2018 (**c**), and Frankel_2017 (**d**) cohorts and tested on the remaining three cohorts separately (filtered RPKM + biological prior knowledge).

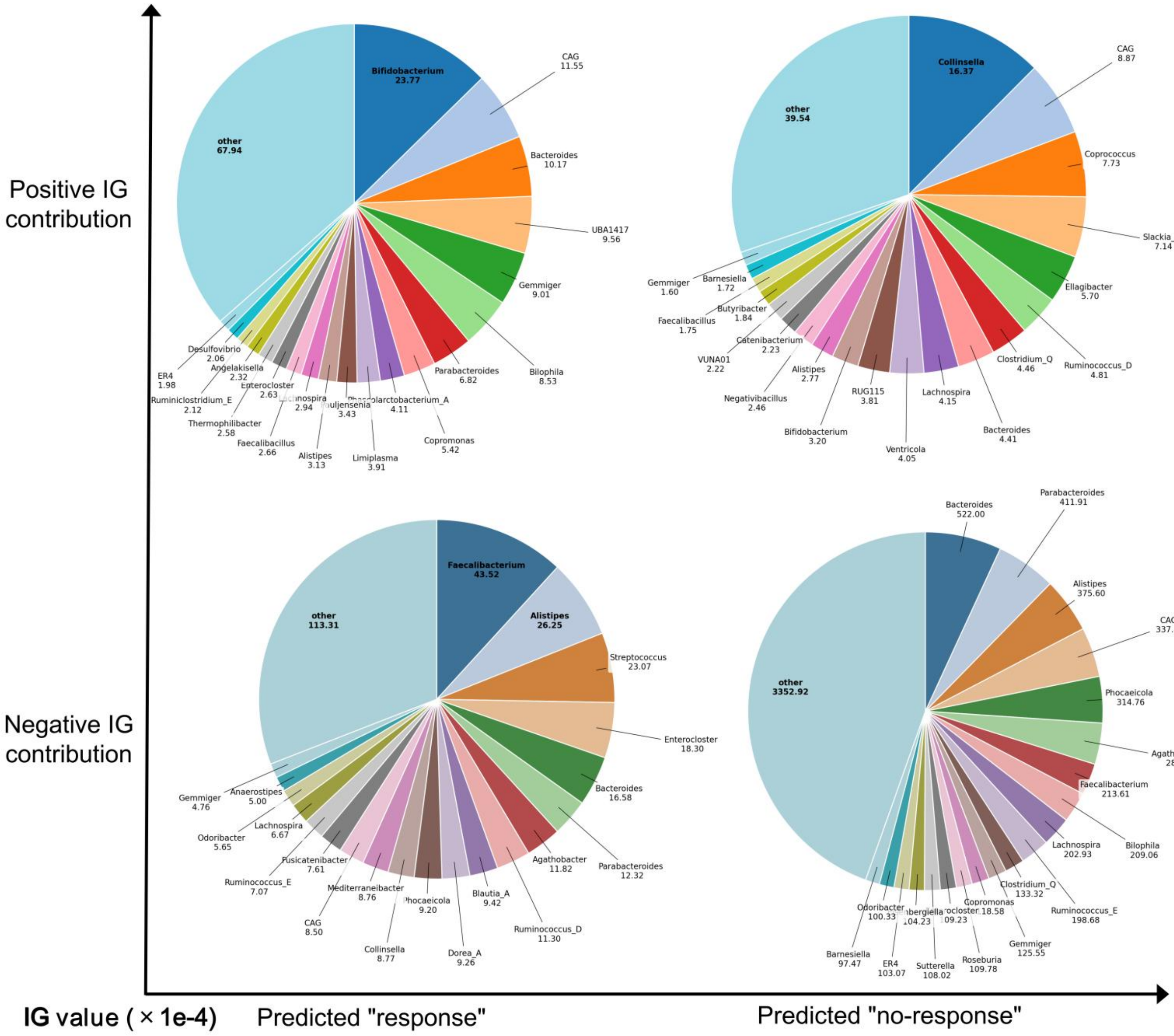

Supplementary Figure 8: The IG attribution analysis on the older cohort using gene-level abundance features (filtered RPKM + biological prior knowledge). IG scores were calculated from the validation sets of five-fold cross-validation, averaging the attribution values from correctly predicted samples across all folds. The feature (contig) name/label was annotated according to the GTDB database. Pie charts showing the cumulative IG contribution aggregated at the genus level, with positive and negative IG values summed separately for responder and non-responder groups, reflecting the overall genus-level influence on ICI therapy response prediction outcomes.

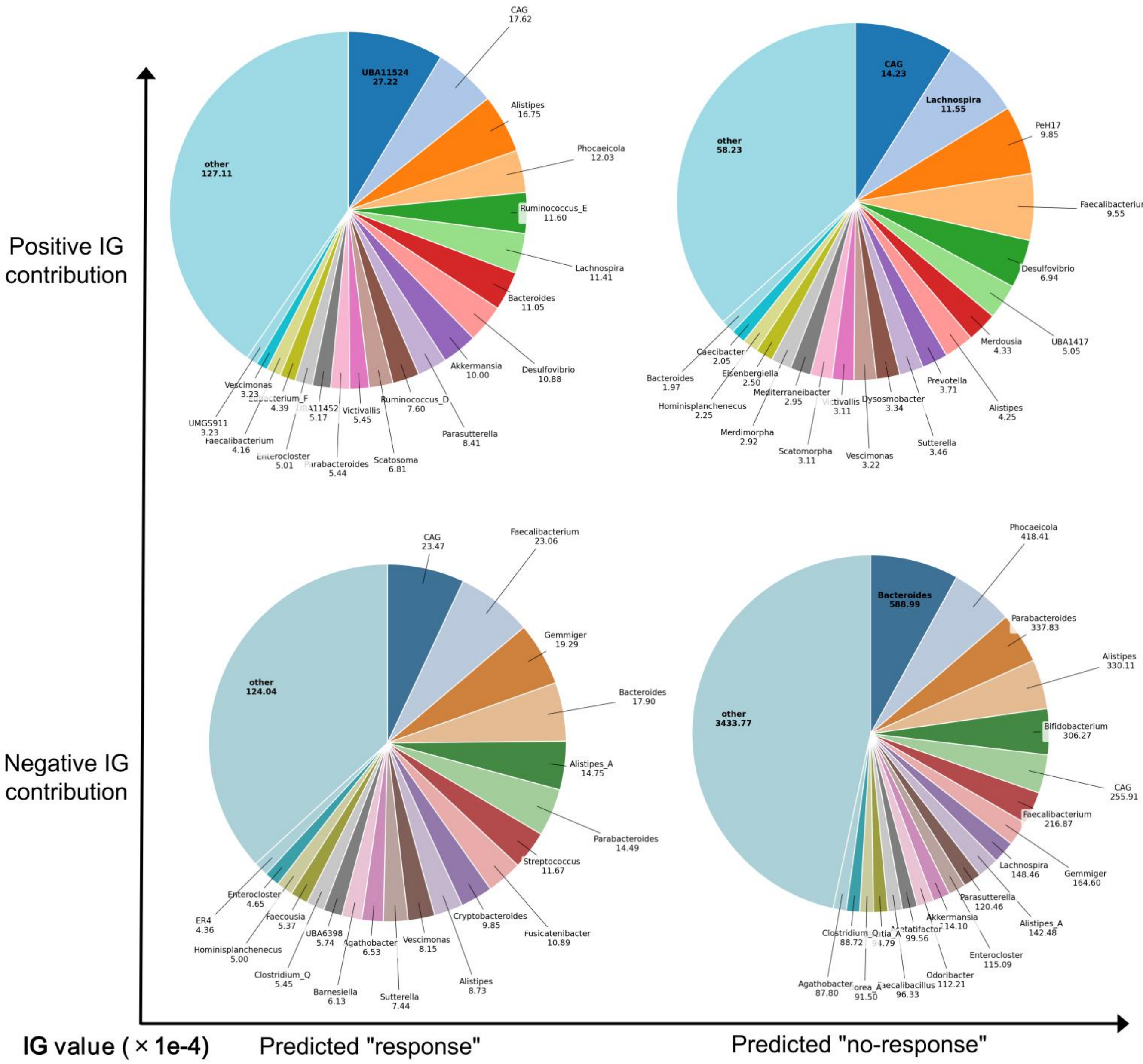

Supplementary Figure 9: The IG attribution analysis on the younger cohort using gene-level abundance features (filtered RPKM + biological prior knowledge). IG scores were calculated from the validation sets of five-fold cross-validation, averaging the attribution values from correctly predicted samples across all folds. The feature (contig) name/label was annotated according to the GTDB database. Pie charts showing the cumulative IG contribution aggregated at the genus level, with positive and negative IG values summed separately for responder and non-responder groups, reflecting the overall genus-level influence on ICI therapy response prediction outcome.

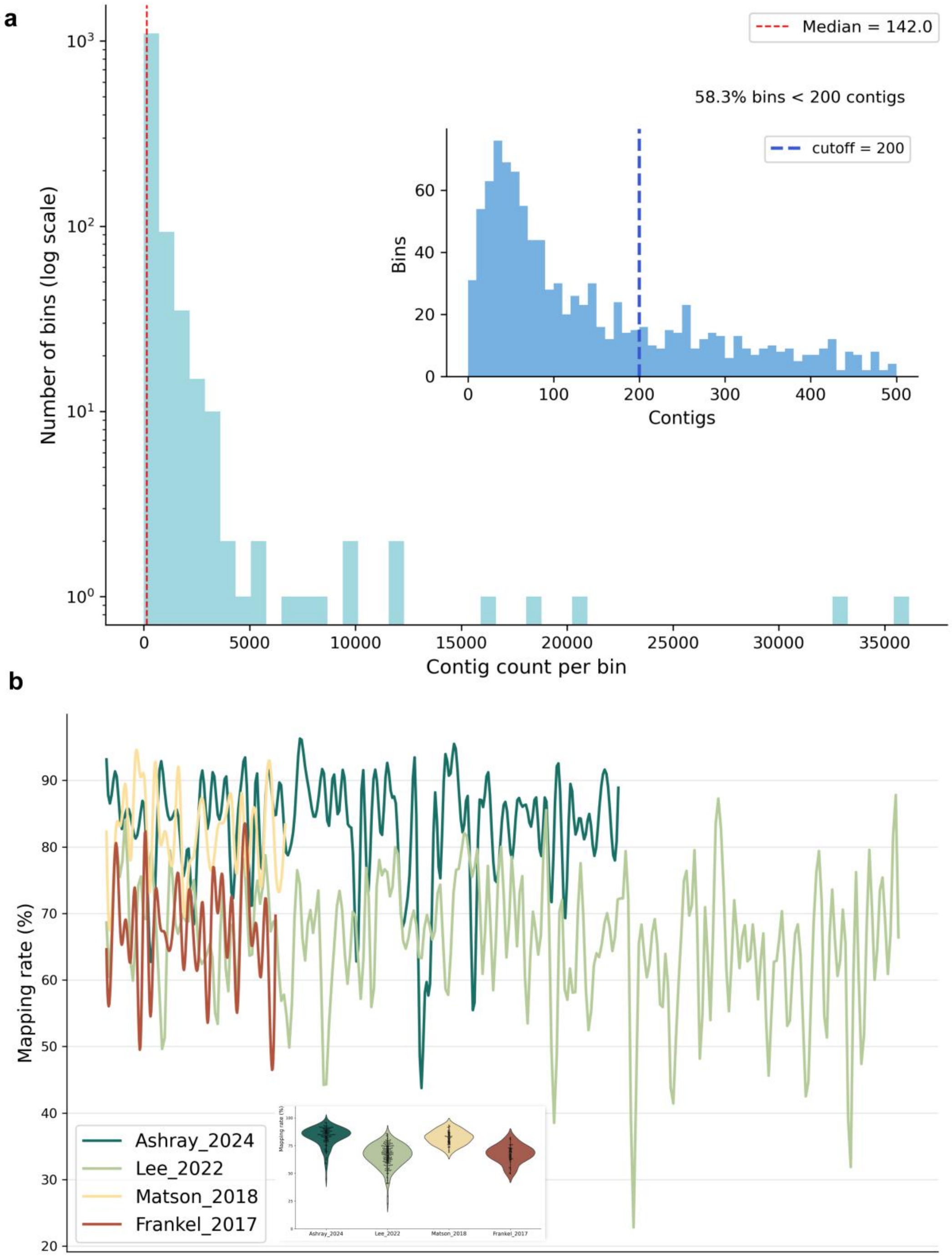

Supplementary Figure 10: a, Contig distribution across genomic bins. We identified 1,269 bins, with contig counts ranging from 3 to 36,143 per bin, reflecting a highly uneven distribution. The median number of contigs per bin was 142, and 58.6% of bins contained fewer than 200 contigs. These results highlight that most bins were relatively compact, whereas only a few bins contained an extremely large number of contigs. **b,** Mapping rate statistics across four cohorts. Mapping rate curves of samples in each cohort, with an inset boxplot showing the distribution of mapping rates. Overall mapping rates were stable across cohorts, ensuring consistency of downstream analyses.

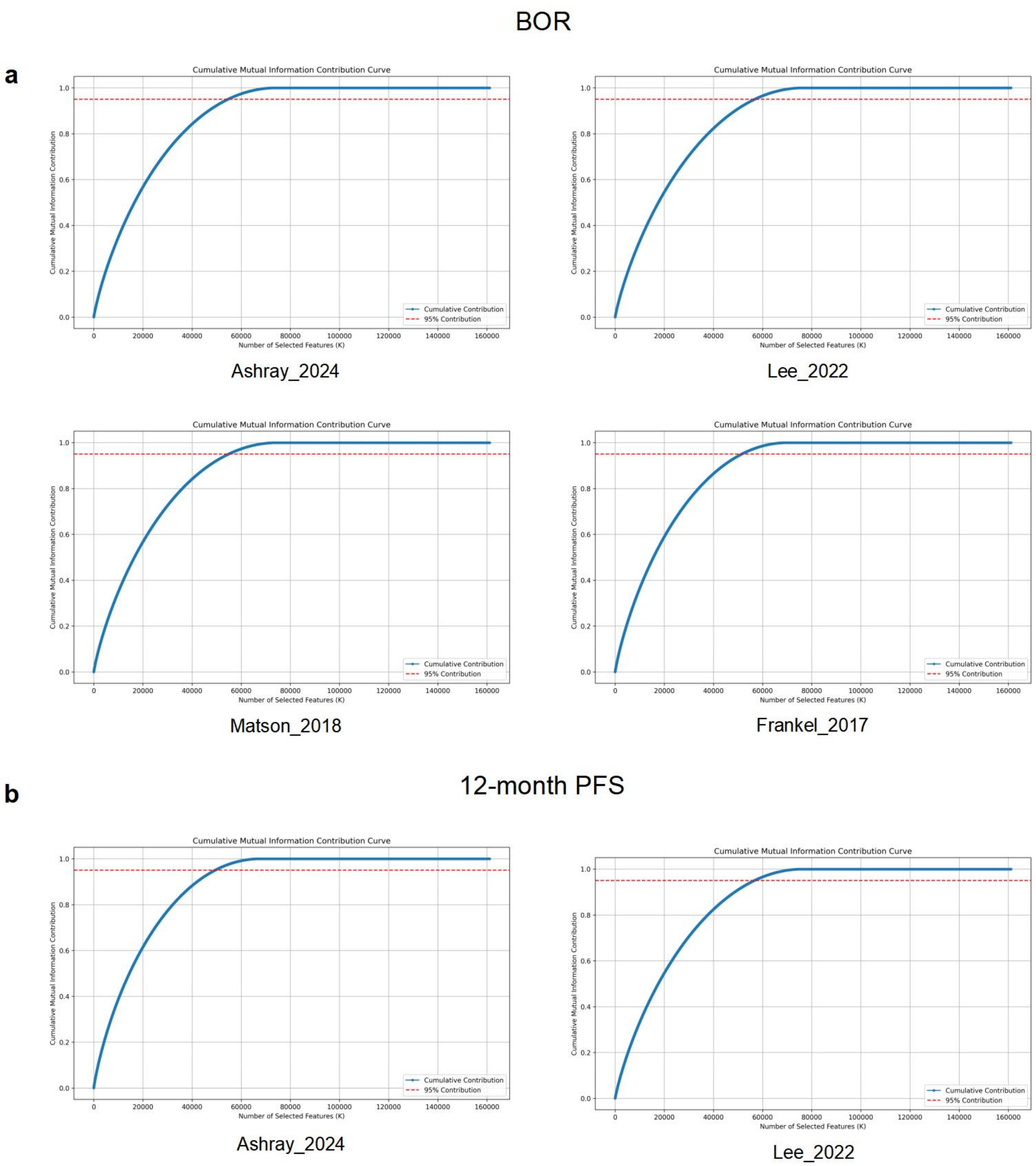

Supplementary Figure 11: Cumulative mutual information contribution curve. The curve rises steeply at the beginning, indicating that a small subset of features carries most of the information; as the number of features increases, the curve gradually flattens. Based on this observation, we selected the top 80,000 features as model inputs to retain essential information while reducing redundancy and noise. **a** shows the curve based on best overall response (BOR), and **b** shows the curve based on 12-month progression-free survival.

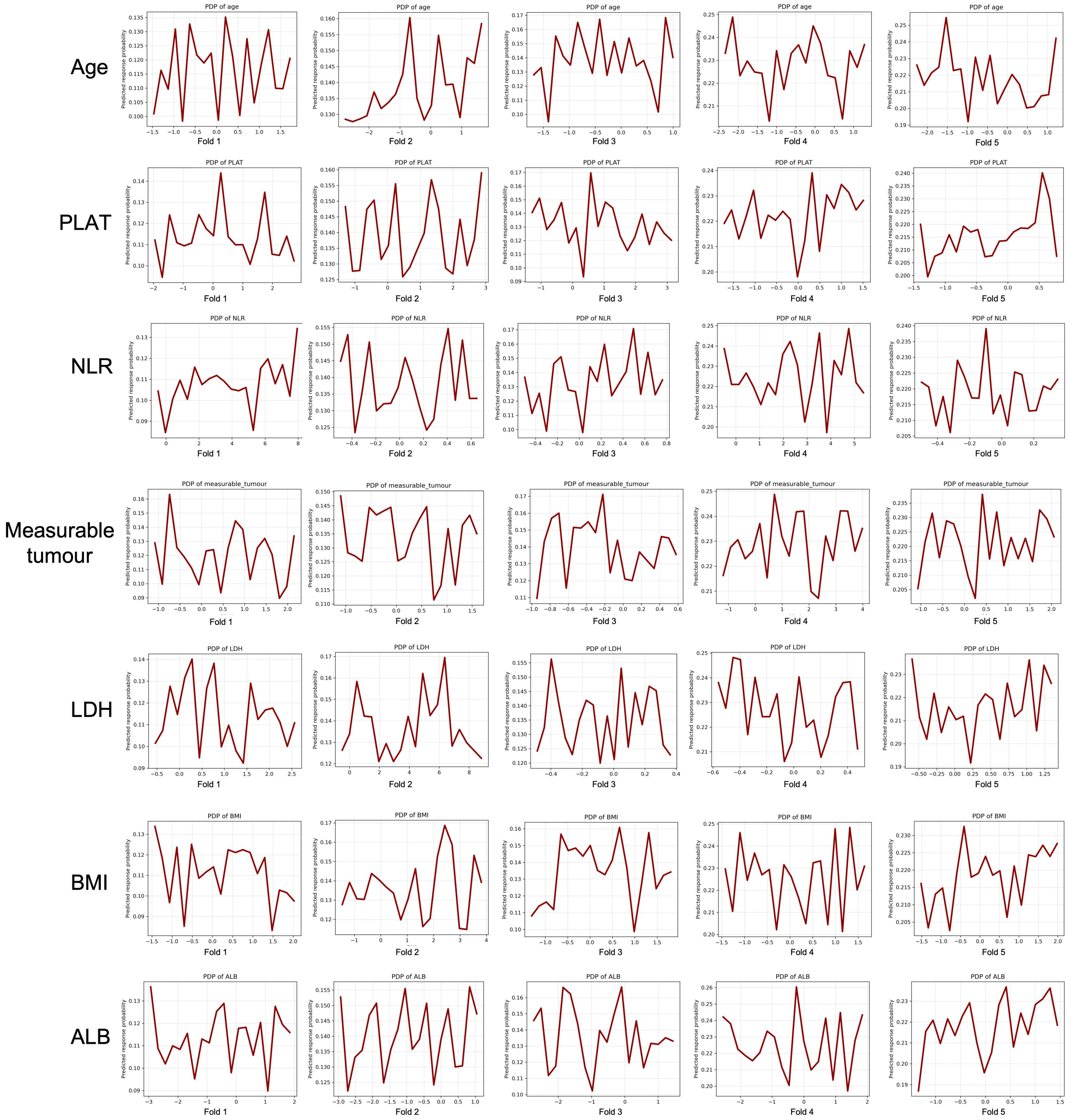

Supplementary Figure 12: Partial dependence plots (PDPs) for seven clinical variables.

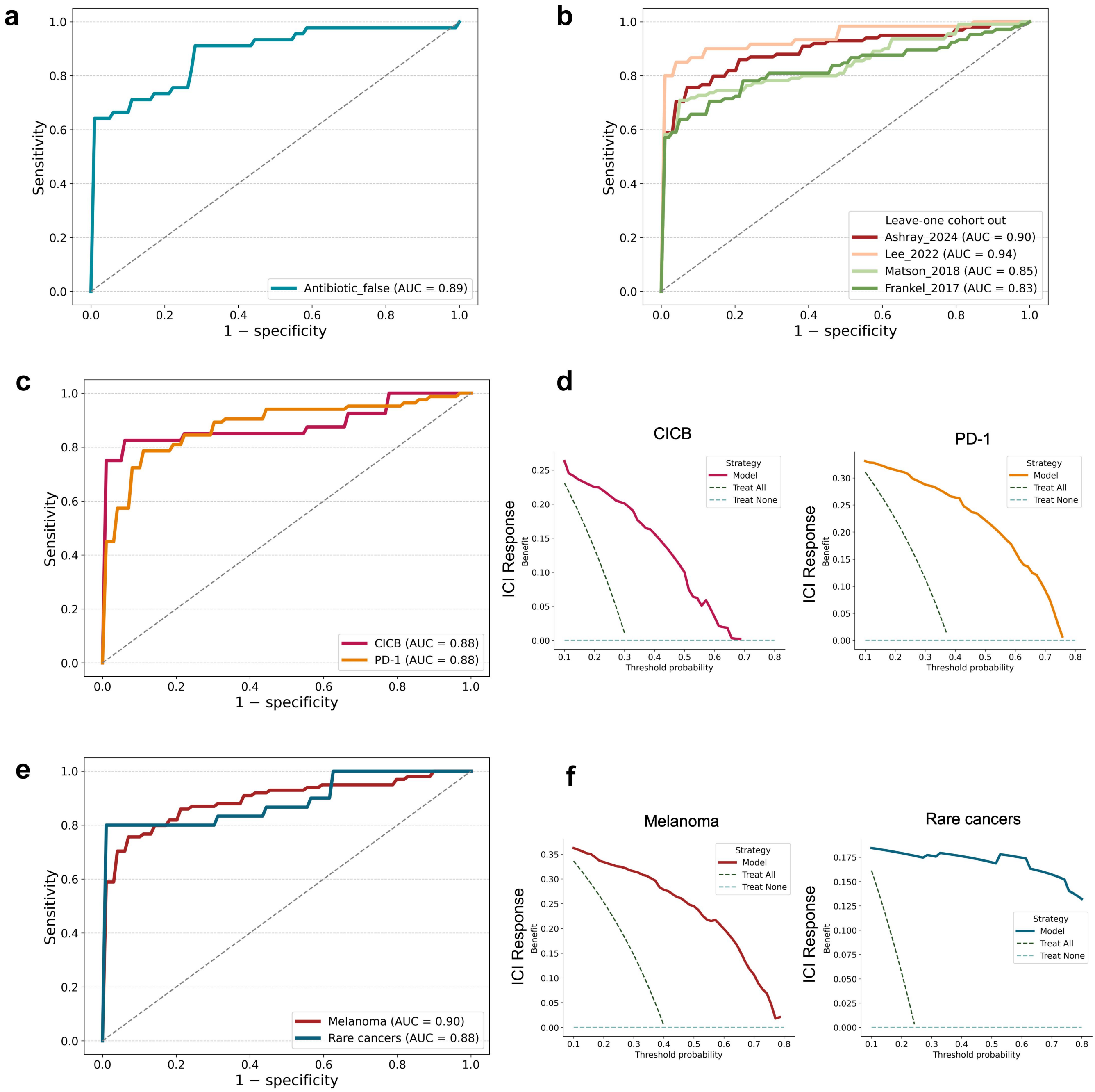

Supplementary Figure 13: Model performance across different stratifications. a, ROC curve for patients without antibiotic exposure. b, ROC curve across leave-one cohort out experiment. c-d, ROC curve (c) and DCA curve (d) across treatment regimens. e-f, ROC curve (e) and DCA curve (f) across cancer types.

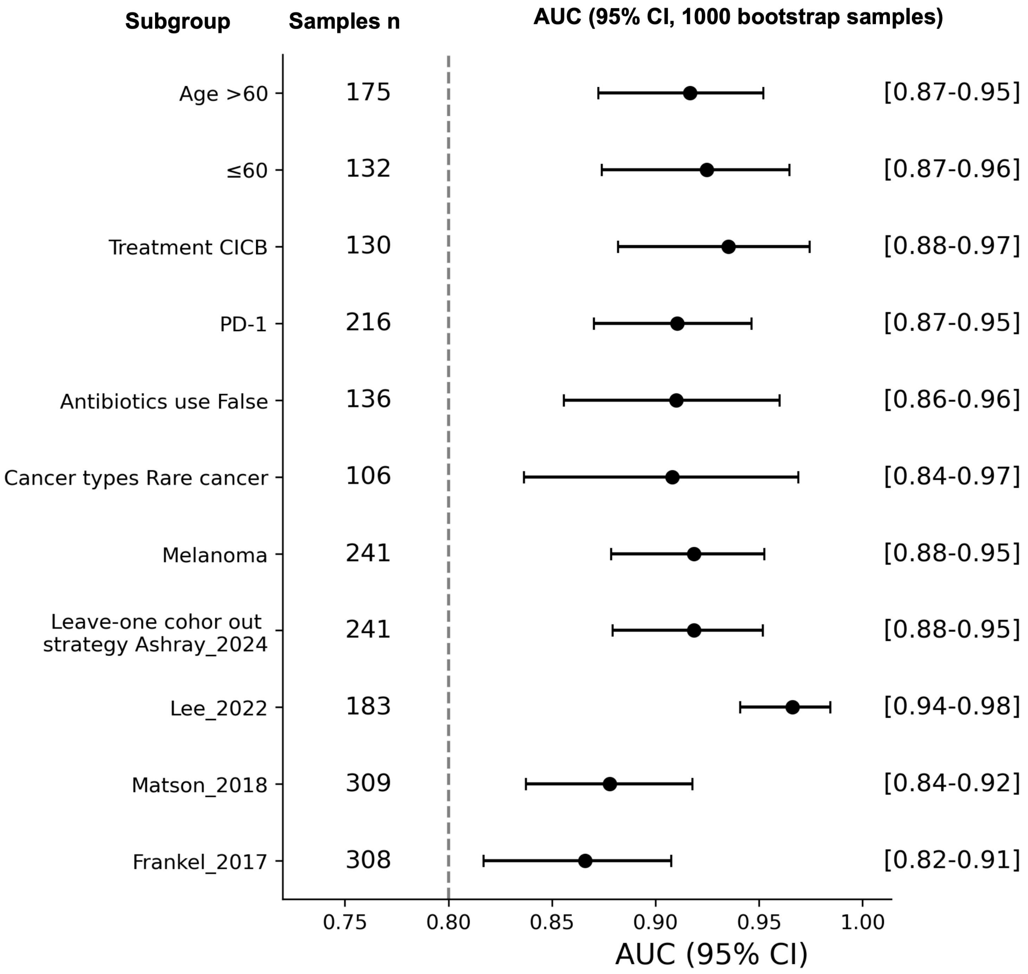

Supplementary Figure 14: Forest plot summarizing model performance across subgroups. Points represent AUC values, with error bars indicating 95% confidence intervals estimated from 1,000 bootstrap samples.

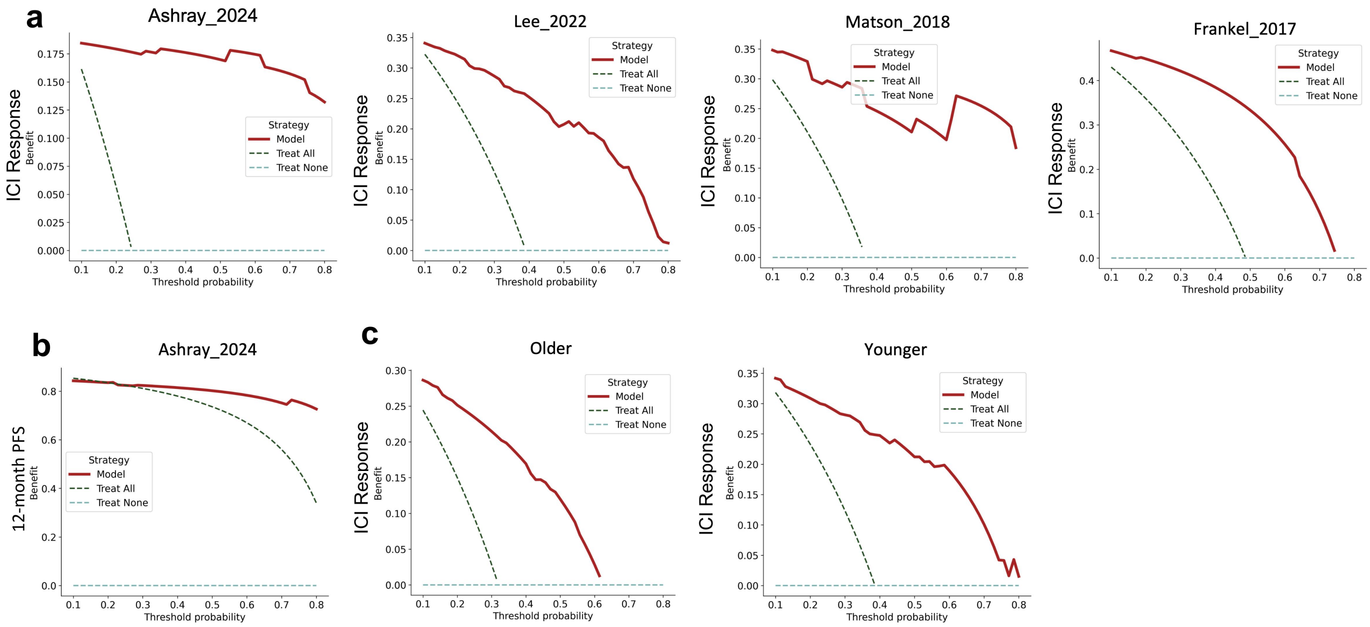

Supplementary Figure 15: DCA curve across treatment regimens and cancer types.

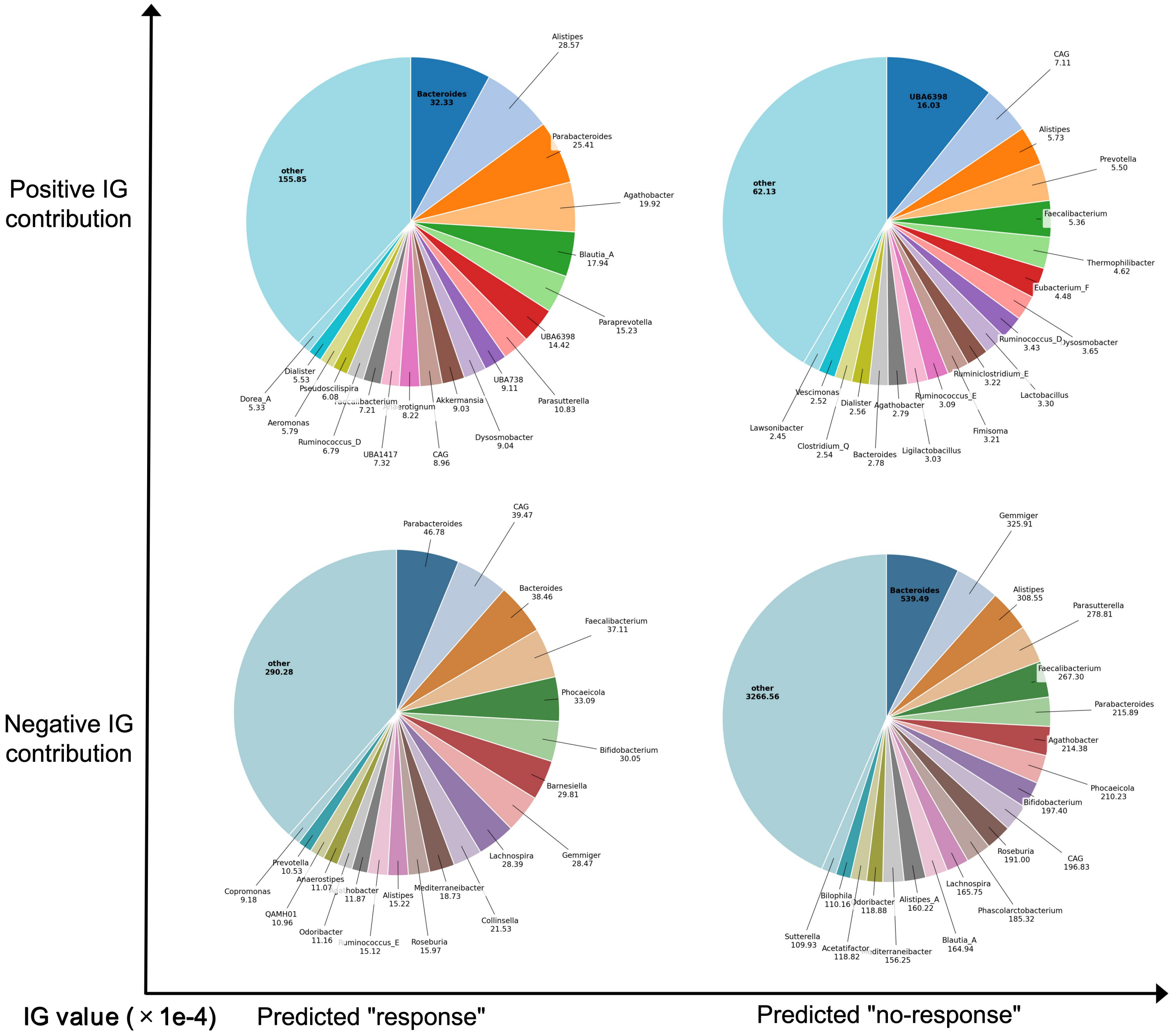

Supplementary Figure 16: The IG attribution analysis on the melanoma group using gene-level abundance features (filtered RPKM + biological prior knowledge). IG scores were calculated from the validation sets of five-fold cross-validation, averaging the attribution values from correctly predicted samples across all folds. The feature (contig) name/label was annotated according to the GTDB database. Pie charts showing the cumulative IG contribution aggregated at the genus level, with positive and negative IG values summed separately for responder and non-responder groups, reflecting the overall genus-level influence on ICI therapy response prediction outcome.

Characteristics	Ashray_2024	Lee_2022	Matson_2018	Frankel_2017
Continent	Oceania	Europe	North America	North America
Country	Australia	UK, Netherlands, Spain	USA	USA
Stool collection kits	OMR-200	LO—TF kits,<br>MA—plain tube,<br>NL—plain tube,<br>LD—OMR-200,<br>BL—OMR-200	EasySampler	NR
DNA extraction kits	FastDNA soil	LO—TF MagMAX,<br>MA—TF MagMAX,<br>LD—TF MagMAX,<br>NL—TF MagMAX,<br>BL—PowerFecal	PowerFecal	Other
Sequencing platforms	Illumina NovaSeq 6000	Illumina NovaSeq 6000	Illumina HiSeq 2000	Illumina HiSeq 2000
Read length (bases per read)	NovaSeq (2 × 151)	NovaSeq (2 × 151)	NextSeq (2 × 151)	NextSeq (2 × 151)
Sequencing depth (clean paired-end reads)				
Minimum	910 M	4.38 M	19.3 M	18.1 M
Median	20.4 M	20.7 M	35.6 M	45.4 M
Maximum	53.8 M	104 M	77.7 M	59.9 M

Supplementary Table 1: Characteristics of cohorts included in the study.

Leave-one cohort out	Accuracy	Precision	Recall	F1	AUC	AUPR
Ashray_2024	0.84	0.78	0.92	0.84	0.90	0.87
Lee_2022	0.84	0.76	0.98	0.84	0.94	0.93
Matson_2018	0.83	0.80	0.76	0.78	0.85	0.80
Frankel_2017	0.82	0.73	0.73	0.73	0.83	0.78
Average	0.83	0.77	0.85	0.79	0.88	0.85

Supplementary Table 2: The average performance of leave-one cohort out.
